# Dual-Mechanism Antimicrobial Peptides from a Nature-Inspired Scaffold

**DOI:** 10.1021/acs.jmedchem.6c00952

**Published:** 2026-07-24

**Authors:** Luisa I. Beyer, Johannes Thoma, Silvana Lord Smits, Annie Justh de Neczpal, Hanna Mårtensson, Julia Leandersson, Maya Tabbaa, Anne Farewell, Åsa Sjöling, Alexandra Stubelius, Alesia A. Tietze

**Affiliations:** † 3570 University of Gothenburg, Department of Chemistry and Molecular Biology, Medicinaregatan 16, Gothenburg 413 90, Sweden; ‡ Wallenberg Centre for Molecular and Translational Medicine, Gothenburg 405 30, Sweden; § Center for Antibiotic Resistance Research in Gothenburg, The University of Gothenburg, Box 100, Gothenburg 405 30, Sweden; ∥ 11248 Chalmers University of Technology, Department of Life Sciences, Kemigården 4, Göteborg 412 96, Sweden

## Abstract

Antimicrobial peptides are promising alternatives to conventional antibiotics, yet systematic strategies to enhance their potency and elucidate their mechanisms of action remain limited. Here, we generated and evaluated a focused library of 20 peptides derived from the lead peptide **L3**. Across clinically relevant pathogens, including *Escherichia coli*, *Klebsiella pneumoniae*, *Staphylococcus aureus*, and *Candida albicans*, several variants showed enhanced antibacterial activity, reducing MIC values to as low as 32 μg/mL (**G2-4**). Additional candidates (**G1-8**, **G2-1**, **G2-2**, **G2-10**) achieved MICs of 64 μg/mL against *E. coli*. Studies in environmental *Escherichia* isolates revealed species-specific susceptibility patterns. Mechanistic investigations demonstrated minimal membrane-lytic activity at concentrations exceeding their MICs, indicating that membrane disruption is not their primary mode of action. In contrast, in vitro transcription/translation assays demonstrated potent inhibition of protein expression. These results demonstrate how targeted sequence refinement can substantially enhance antimicrobial potency while modulating interactions with bacterial membranes and the transcription/translation machinery.

## Graphical abstract

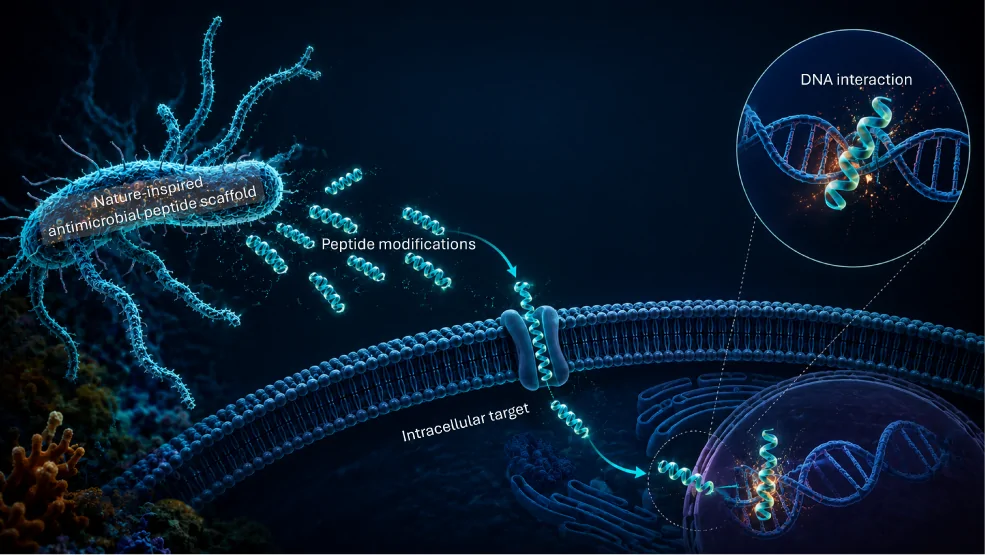

## Introduction

Antibiotic and antimicrobial resistance (AMR) is one of the most severe public health challenges of our time, demanding urgent action against resistant bacterial strains.^1,2^ As resistance levels rise, conventional medications lose effectiveness, rendering infections increasingly difficult or even impossible to treat. One promising strategy for combating AMR is to develop new compounds that overcome bacterial resistance mechanisms. Among these, antimicrobial peptides (AMPs) have gained attention because of their diverse mechanisms of action, which may reduce the likelihood of resistance development.^3,4^

Traditionally, AMPs are known for their nonspecific, membrane-lytic bactericidal activity. Most act by disrupting bacterial membranes through transmembrane pore/channel formation or extensive membrane rupture, ultimately causing lysis and cell death.^5,6^ However, this membrane-lytic activity often leads to toxicity in host cells, as exemplified by polymyxins, which have substantial nephrotoxic and neurotoxic effects.^7^

Consequently, research interest has shifted toward AMPs that act through mechanisms beyond membrane disruption. Dual-mechanism AMPs, capable of both membrane permeabilization and intracellular targeting, have gained scientific interest.^8,9^ Current efforts focus on designing peptides that also target intracellular components, thereby complementing membrane activity to enhance bactericidal efficacy and reduce the likelihood of resistance development.^6,10^ For example, the tryptophan- and proline-rich peptide Indolicidin permeabilizes bacterial membranes without causing complete lysis. Its primary mode of action involves binding to DNA, inhibiting DNA synthesis, and inducing bacterial filamentation.^11,12^ Similarly, the histone-derived peptide Buforin II is a cationic, amphipathic AMP that penetrates bacterial membranes via a central proline “hinge” and binds nucleic acids of DNA or RNA, thereby interfering with the bacterial transcription and translation machinery.^13,14^ Another marine-derived peptide, L3, was previously discovered by us, which induces membrane depolarization in *E. coli* without causing membrane rupture or cell lysis. Its antimicrobial activity primarily involves targeting bacterial DNA and disrupting key information-processing pathways.^15,16^

The concept of dual-mechanism AMPs has inspired the design of novel antimicrobial strategies, including hybrid peptides and peptidomimetic antibiotics that integrate both membrane-active and intracellular-targeting motifs.^17–19^ Rational peptide design in this context focuses on balancing amphipathicity to achieve efficient, multistep bacterial killing with minimal host toxicity.^5,20,21^ Nevertheless, even small variations in structural and sequential features, such as net charge, hydrophobicity, or helicity, can significantly influence antimicrobial potency. Systematic modifications in amino acid composition and peptide folding are therefore essential to fine-tuning AMPs for both effective membrane penetration and precise intracellular targeting.^22^

In this study, we aim to rationally design the previously discovered marine-derived AMP **L3** ^15,16^ by introducing sequence modifications aimed at improving antimicrobial activity and exploring how amino acid composition and secondary structure influence antimicrobial activity. The designed peptides were synthesized and evaluated for antimicrobial activity against a panel of microorganisms, including Gram-positive and Gram-negative bacteria and yeast strains. Mechanistic analysis was performed to assess the dual action as membrane permeabilization/disruption and possible interference with intracellular transcriptional and/or translational processes.

## Results and Discussion

### Rational Design of Peptides

In an initial step, peptide **L3** was employed as the starting sequence and subjected to systematic modification guided by physicochemical properties and predicted conformational effects of individual amino acids with the goal of enhancing membrane interactions, cellular permeability, and DNA binding (Figure 1).

**Figure 1:**
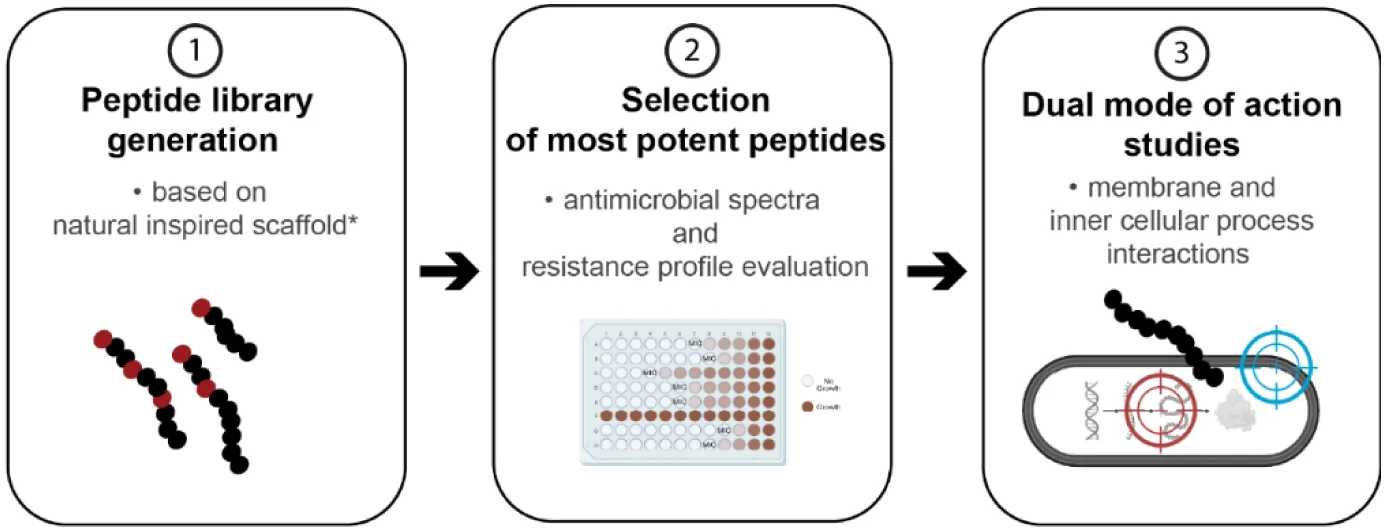
Schematic strategy of the study. *Nature-inspired peptide sequence L3 (Beyer et al., 2024, 2026).^15,16^

The first-generation analogues (**G1-1** to **G1-8**) incorporated the following substantial alterations to the parent L3 sequence. The initial modification involved replacing nitrotyrosine (Tyr­(NO_2_)) with tyrosine (Tyr) (Figure 2, peptide **G1-1**). Tyr­(NO_2_) has a phenolic p*K*_a_ close to physiological pH, allowing its hydroxyl group to deprotonate and contribute to local electrostatic variation.^23^ Its strong electron-withdrawing nitro substituent increases the negative character of this residue. Substitution with Tyr removes this nitro group, thereby reducing the local electron-withdrawing effect and decreasing the negative charge contribution at this position. Stereochemical influences on peptide conformation were examined by altering the D-amino acid configuration to L-amino acids. The lead peptide L3 contains four D-amino acids, which are expected to play a key role in defining peptides’ conformation and stability. To determine whether these D-residues are essential for activity, or whether replacing them with their l-enantiomers could alter secondary structure relevant to membrane engagement or DNA binding, the two d-Val amino acids in the L3 peptide were substituted by l-Val in peptide **G1-2**, and all four D-amino acids were changed to L-amino acids in peptides **G1-3** to **G1-8**. In addition to these changes, Tyr­(NO_2_) was replaced with leucine (Leu) (**G1-4**) to investigate if the incorporation of a hydrophobic amino acid influences the activity. In peptides **G1-5** and **G1-6**, hydroxyproline (Hyp) was introduced at two independent positions to assess conformational constraints, as its rigid pyrrolidine ring and fixed hydroxyl orientation restrict local backbone flexibility and might modify hydrogen-bonding patterns, thereby influencing global peptide architecture (**G1-5** and **G1-6**). Two additional modifications targeted the positively charged amino acids in the L3 peptide to modulate local charge distribution while maintaining the overall net positive charge. The two ornithine (Orn) residues were replaced with lysines (Lys) (**G1-7**), whose extended side chain provides increased flexibility. In peptide **G1-8**, the two Orn and the Lys were substituted with three arginines (Arg) (**G1-8**). Guanidinium groups support extensive charge delocalization and form strong hydrogen bonds. These features are expected to enhance electrostatic interactions with anionic targets, including bacterial membranes and DNA.^24^

**Figure 2:**
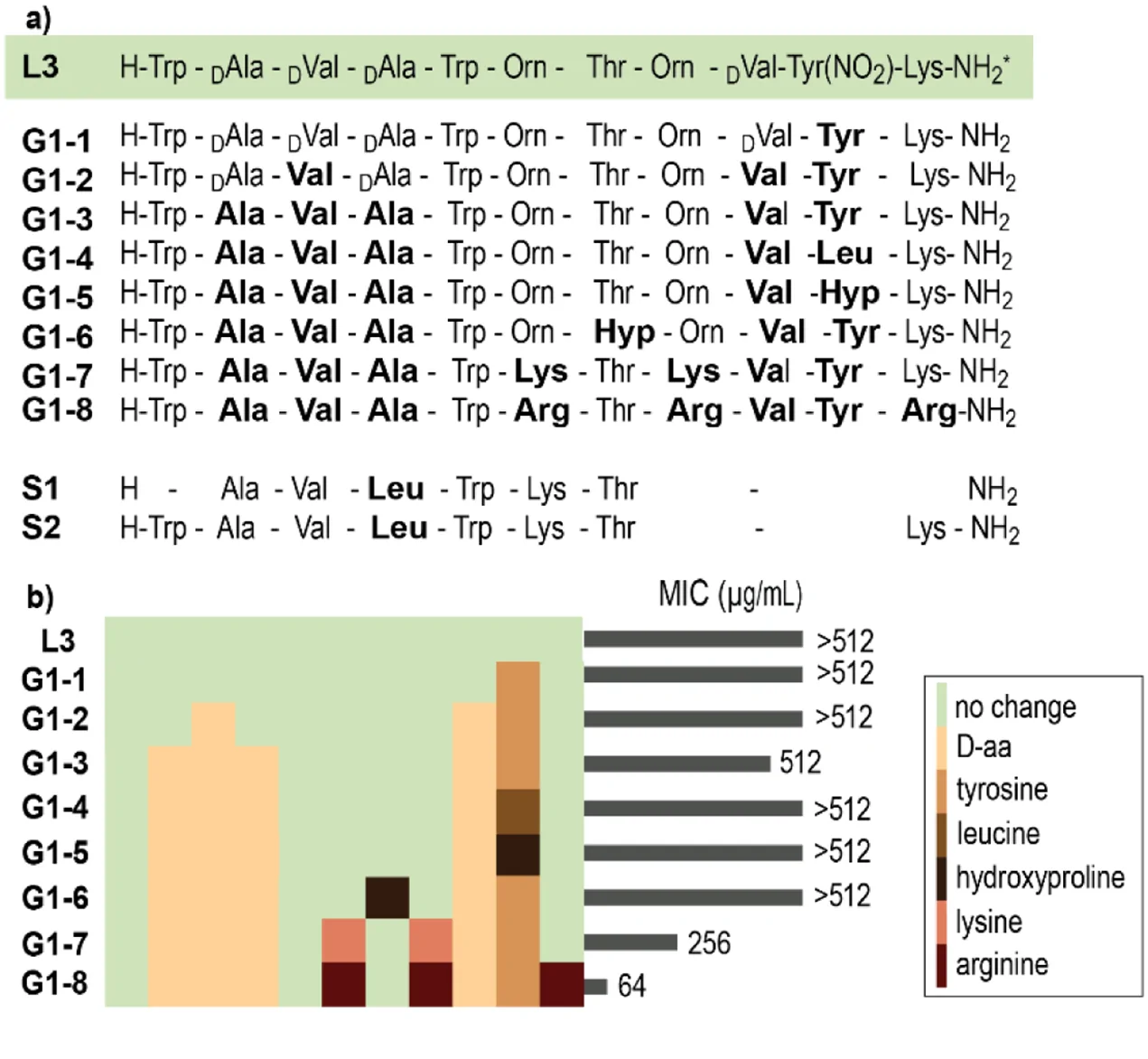
First-generation peptide sequences and their bioactivities. a) Amino acid sequences highlighting the changes of amino acids in bold. b) Heatmap showing the peptides’ antimicrobial activity, measured as MIC against *E. coli* CCUG 17620. * Lead sequence L3 is from Beyer et al.^15,16^

All rationally designed peptides were chemically synthesized and tested for antibacterial activity against a recommended reference strain for antibiotic susceptibility testing, *E. coli* (CCUG 17620). The MIC values are shown in Figure 2b. In comparison to L3, which showed MIC > 512 μg/mL, all peptides containing D-amino acids, **G1-1** and **G1-2**, showed a marked loss of antimicrobial activity, with MIC > 512 μg/mL. In contrast, some peptides composed solely of L-amino acids possess antimicrobial activity, suggesting that substituting D-amino acids with their L-counterparts enhances antimicrobial activity. Also, introducing Hyp at a single position (**G1-5** or **G1-6**) did not confer antimicrobial activity. Peptides **G1-3**, **G1-7**, and **G1-8** showed stepwise increases in antibacterial activity, with MIC values of 512, 256, and 64 μg/mL, respectively. The difference among the three peptides lies in the composition of the positively charged amino acids at positions 6, 8, and 10. The lead **L3** sequence and peptide **G1-3** contain Orn and Lys. Substitution of Lys in all positions improved the antimicrobial activity 2-fold (**G1-7**). Interestingly, another 4-fold increase in activity was obtained with three Arg residues at these positions (**G1-8**).

A second-generation peptide library was designed from the lead sequence **G1-8**, consisting of systematic single amino acid substitutions aimed at assessing the influence of hydrophobicity, aromaticity, charge distribution, and conformational constraints on antimicrobial activity and is summarized in Figure 3.

**Figure 3:**
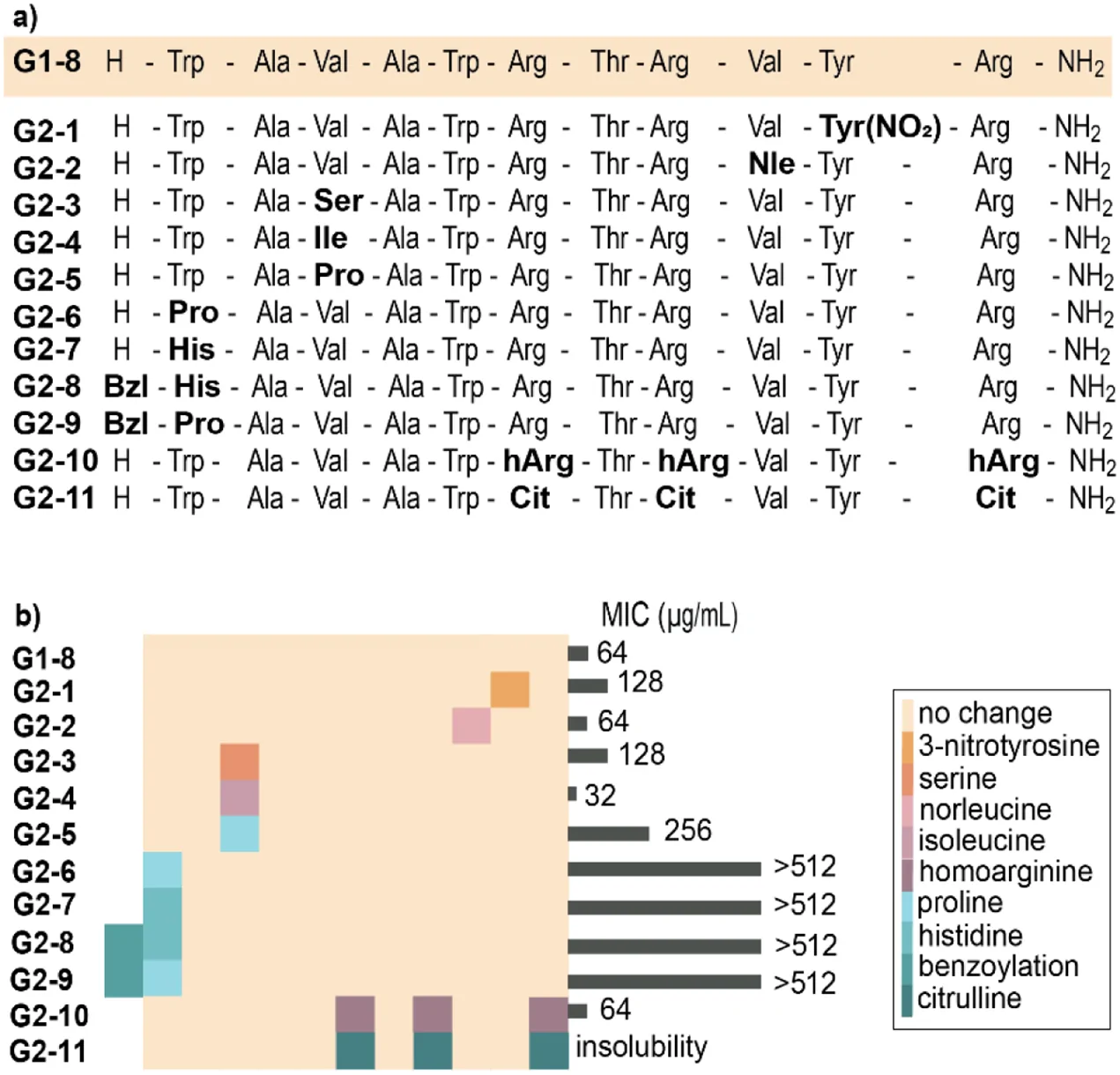
Second-generation peptide sequences and their bioactivities. a) Amino acid sequences highlighting the changes of amino acids in bold. b) Heatmap showing the peptides’ antimicrobial activity, measured as MIC against *E. coli* ATCC 25922.

Substitutions affecting hydrophobicity and local structure at position 9 or 10 involve replacing Tyr with Tyr­(NO_2_) (**G2-1**) or norleucine (Nle) (**G2-2**). Tyr­(NO_2_) produced a moderate decrease in potency (128 μg/mL), whereas substitution of Tyr with Nle did not change the activity, indicating that enhanced hydrophobicity at this position is well tolerated. At position 3 (**G2-3** to **G2-5**), replacement of valine (Val) with serine (Ser) resulted in a modest loss of activity, while the introduction of isoleucine (Ile) improved potency to an MIC of 32 μg/mL (G2-4). Substitution with proline (Pro) markedly reduced activity, reflecting proline’s contribution to disruption of local secondary structure and amphipathicity.^25^ Replacing the N-terminal tryptophan (Trp) with Histidine (His), Pro, or their benzylated analogues resulted in a drastic loss of antimicrobial activity. Since Trp plays an essential role in membrane engagement through hydrophobic and π interactions, the removal or alteration of this aromatic residue strongly impairs peptide function.^26^ Modifying the amino terminus, Trp to Pro (G2-6), His (**G2-7**), Bzl-His (**G2-8**), or Bzl-Pro (**G2-9**) leads to inactive peptides with MICs > 512 μg/mL. Substitution of Arg with homoarginine (hArg) (**G2-10**) retained activity comparable to the lead peptide (MIC 64 μg/mL), demonstrating that a modest extension of the side chain without a change in charge is tolerated. In contrast, replacement with citrulline (Cit) (**G2-11**) abolished the positive charge and caused peptide insolubility, preventing reliable MIC determination.

Overall, the two above-described rounds of sequence design resulted in a total of five peptides with promising antibacterial activity with MIC values between 32 and 128 μg/mL, which were further investigated: **G1-8**, **G2-1**, **G2-2**, **G2-4**, and **G2-10**.

### Secondary Structure Analysis

To experimentally assess whether the sequence modifications introduced during peptide design influence secondary structure, the most potent peptides **G1-8**, **G2-1**, **G2-2**, **G2-4**, and **G2-10** were analyzed by circular dichroism (CD) spectroscopy and solution NMR. CD spectra were recorded in water and in 2,2,2-trifluoroethanol (TFE), which was used as a membrane-mimetic, structure-promoting environment (Figure S39, Table S2). {Prasad, 2024 #481} In aqueous solution, the selected peptides displayed low helical content, dominated by β-structure and unordered structural contributions. **G1-8**, **G2-1**, and **G2-4** showed no detectable helical content in water, while **G2-2** and **G2-10** displayed only modest helicity of approximately 10%. In contrast, a pronounced increase in helical content was observed in TFE for all five peptides, indicating that peptides remain relatively flexible in aqueous solution and become more structured in a membrane-mimicking environment. Thus, the selected peptides possess a tendency to adopt ordered conformations in a membrane-mimicking environment.

Solution NMR further supported this environment-dependent structural behavior. Comparison of the line representation of NMR structures revealed that peptides share a compact, bent backbone but flexible conformations and side-chain orientation (Figure 4a). The superposition of the 20 lowest-energy conformers (Figure S40) showed good local structural alignment in the central peptide region, while C- and N-terminal and side-chain orientations remained highly flexible, consistent with the tendency observed by CD. The degree of flexibility differed between the peptides. The backbone RMSD calculated over the 20 best structures was lowest for the peptide with the strongest antimicrobial activity, **G2-4**, at 0.94 Å. It was followed by **G2-10** with 1.26 Å, **G1-8** with 1.64 Å, **G2-2** with 2.12 Å, and finally **G2-1** with 2.23 Å. Thus, G2-4 displayed the more well-defined solution ensemble among active peptides, suggesting that the Val to Ile mutation may stabilize a favorable backbone arrangement. The van der Waals surface representation (Figure 4b) showed that the peptides display spatially separated cationic/polar and hydrophobic regions, consistent with amphipathic organization. Such an arrangement is suggested to facilitate initial membrane interaction without membrane lysis and subsequent intracellular activity. Importantly, the structural data indicate that antibacterial potency is not governed by solely helicity/ordered structure adaptation. The improved activity of the most potent peptide **G2-4** likely reflects an optimized combination of several parameters, such as inducible secondary structure, side chain chemical properties, charge distribution, amphipathic properties, and target engagement.

**Figure 4:**
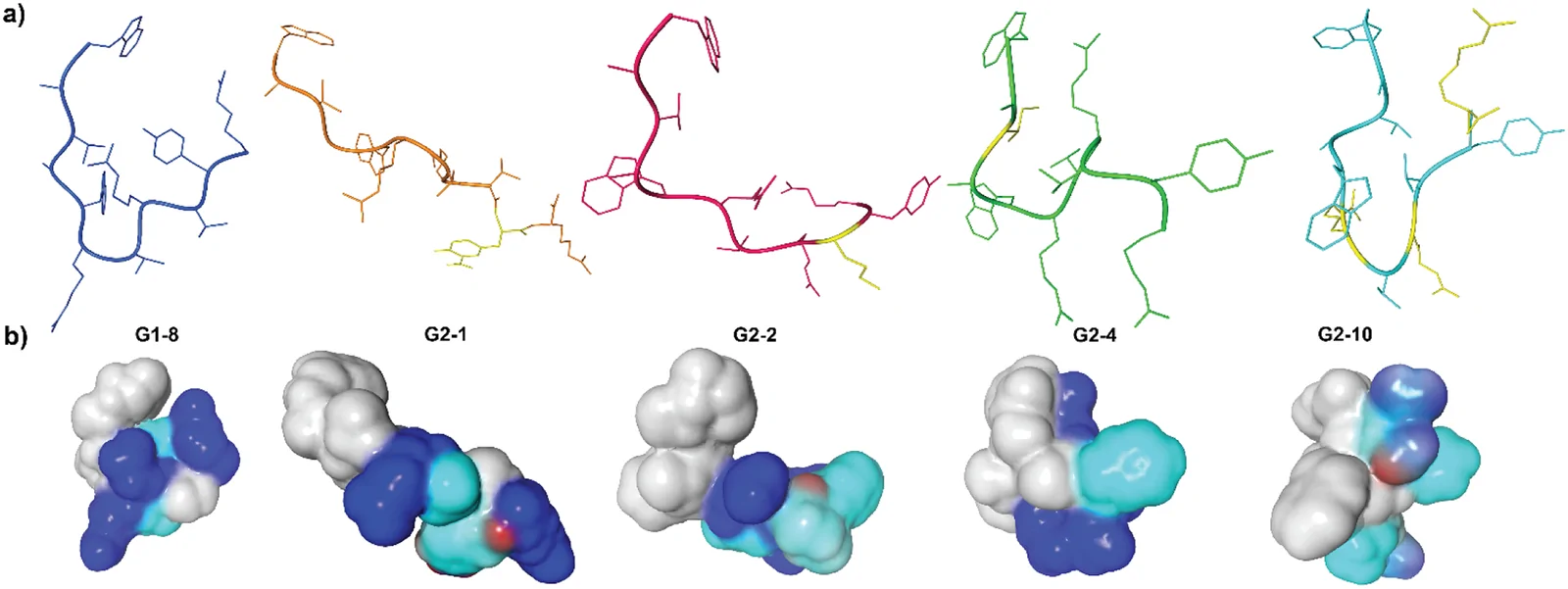
Solution NMR structures of peptides **G1-8**, **G2-1**, **G2-2**, **G2-4**, **G2-10**. a) Representative solution NMR structures of **G1-8**, **G2-1**, **G2-2**, **G2-4**, and **G2-10**, shown from left to right as backbone/side-chain line representations. Structures were calculated from NOE-derived distance restraints and refined in water. b) Corresponding van der Waals surface representation of the same peptides, colored according to amino acids’ physicochemical properties: polar residues in cyan, basic residues in blue, acidic residues in red, and nonpolar residues in gray. The surface representations illustrate the spatial distribution of cationic, polar, and hydrophobic regions within the optimized peptide analogues.

### Broad Bioactivity Evaluation

The antimicrobial spectrum of the five selected peptides (**G1-8**, **G2-1**, **G2-2**, **G2-4**, **G2-10**) was further evaluated against additional microorganisms, including the Gram-negative *Klebsiella pneumoniae* (*K. pneumoniae*), the Gram-positive *Staphylococcus aureus* (*S. aureus*), and the yeast *Candida albicans* (*C. albicans*) (Table 1).

**Table 1:** Activity against Four Recommended Reference Strains for Antibiotic Susceptibility Testing

Peptide	*E. coli* CCUG 17620 [μg/mL]	*K. pneumoniae* CCUG 28450 [μg/mL]	*S. aureus* CCUG 1800T [μg/mL]	*C. albicans* CCUG 31028 [μg/mL]
G1-8	64	128	128	64
G2-1	128	256	128	64
G2-2	64	128	128	64
G2-4	32	64	128	64
G2-10	64	128	128	128

Across the panel of microorganisms, peptide **G2-4** showed the most vigorous overall activity, with the lowest MIC values, particularly against *E. coli* (32 μg/mL) and *K. pneumoniae* (64 μg/mL). Peptides **G1-8**, **G2-2**, and **G2-10** had comparable activity profiles, each showing similar potency against *E. coli* (64 μg/mL) and reduced efficacy against *K. pneumoniae* and *S. aureus* (128 μg/mL). Among these, peptide **G1-8** and **G2-2** maintained better activity toward *C. albicans* (64 μg/mL), whereas peptide **G2-10** had the weakest yeast activity within this group (128 μg/mL).

### Evaluation of Antibiotic Resistance Using Phenotypic Assays

To assess potential associations between peptide activity and specific resistance mechanisms, strains originating from environmental waters and wastewater treatment plants in Sweden, previously characterized for their antibiotic resistance genes, conjugative plasmids, virulence, and phenotypic resistance profiles, were selected. These were represented by seven *E. coli* and three *E. marmotae* isolates (Table 2). The collection represents a broad range of relevant pathogenic isolates with diverse resistance phenotypes, including multidrug-resistant strains and those with minimal or no detectable phenotypic resistance.^27^ Again, here we proceeded with five selected peptides (**G1-8**, **G2-1**, **G2-2**, **G2-4**, and **G2-10**).

**Table 2:**
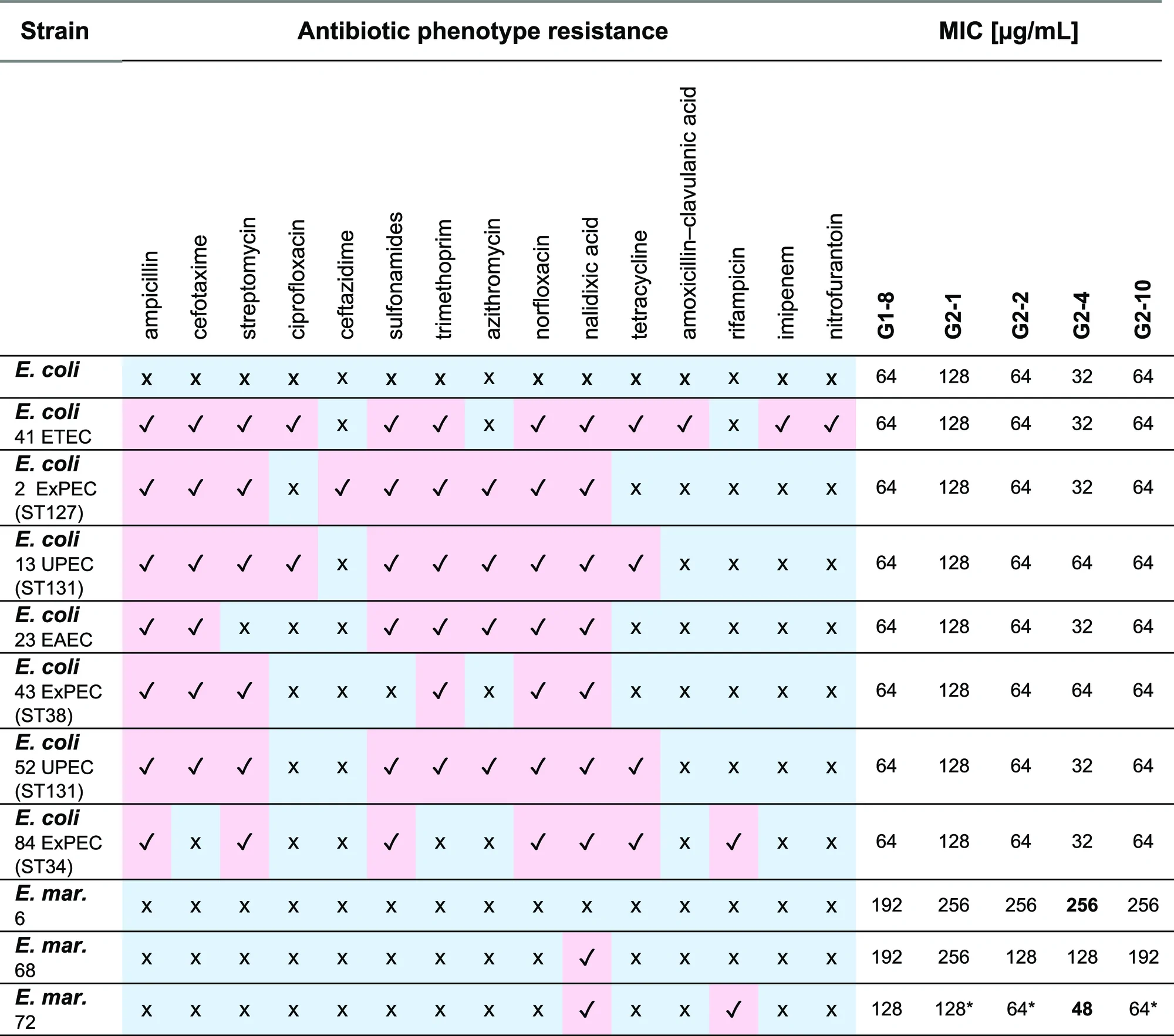
Environmental *Escherichia* spp. Strains with Different Pathotypes and Resistant Genes^a^

a Measurements performed in duplicates, * only single measurement. ETEC = Enterotoxigenic *E. coli*, ExPEC = extraintesitnal *E. coli*, UPEC = uropathogenic *E. coli*. ST = Multi-Locus Sequence Type, ST type ST131 is a globally disseminated urinary tract and bloodstream infection pathogen known to be increasingly multiresistant. ST38, ST34, and ST127 belong to globally disseminated pathogenic clones, while the other isolates belong to less common ST types. Pink indicates resistance, and blue indicates absence of resistance.

Interestingly, for all five selected peptides (**G1-8**, **G2-1**, **G2-2**, **G2-4**, **G2-10**), MIC values were not affected by the resistance profiles or virulence factors of the bacterial strains. Despite their phenotypic resistance to a palette of 15 conventional antibiotics, such as ampicillin, streptomycin, and tetracycline, the resistant isolates were as susceptible to the peptides as nonresistant strains (Table 2). This indicates that even extensive multidrug resistance did not influence susceptibility to the peptides. This suggests that a mode of action of peptides is distinct from, and not affected by, established antibiotic resistance mechanisms. Across the three *E. marmotae* strains (6, 68, and 72), the antimicrobial activity of the five selected peptides showed no indication of being hindered by the bacterial resistance profiles. In fact, MIC values tended to improve slightly in the more resistant isolates. In the fully susceptible strain 6, MICs ranged from 192 to 256 μg/mL, whereas strain 68, which is resistant to nalidixic acid, showed similar or modestly lower MICs (128–256 μg/mL). This trend was even more pronounced in strain 72, which carries resistance to both nalidixic acid and rifampicin, where all peptides displayed their highest potency, with MICs decreasing to 48–128 μg/mL. These observations indicate that the peptides retain, and in some cases enhance, their activity against antibiotic-resistant *E. marmotae*, underscoring that their mechanism of action is distinct from the intracellular targets of nalidixic acid and rifampicin, DNA gyrase^28^ and RNA polymerase,^29^ but does not exclude this target in general.^30^ Moreover, the increased susceptibility of multidrug-resistant strains suggests that resistance-associated alterations, such as changes in membrane composition or permeability, may further enhance the effect of peptides on these bacteria and warrant further investigation.

The divergence in the effects of peptides on *E. coli* and *E. marmotae* strains likely reflects species-specific differences in cell envelope structure and function. These results highlight that the impact of antibiotic resistance on peptide susceptibility can vary substantially between closely related species, depending on how resistance is physiologically developed, and provide means to study intrinsic peptide resistance further.

As the next step, we aimed to understand how sequential modifications influence the peptides’ mode of action. First, the membrane activity was studied.

### Peptides/Membrane Interaction

To evaluate the membrane activity of the selected peptides, a panel of *E. coli* variants with defined outer-membrane or LPS alterations^31^ was tested (Table 3). *E. coli* MG1655 is a standard K-12 laboratory strain and serves as the wild-type reference. *E. coli* strain MG1655wbbL^+^ expresses the rhamnosyltransferase (WbbL) involved in the LPS biosynthetic pathway, which restores the outer membrane O-antigen otherwise lacking in K-12 strains. Consequently, it has an increased resistance to membrane-targeting agents.^32^ MG1655ΔrfaC carries a deletion in an essential LPS core gene, resulting in a deep-rough phenotype with high membrane permeability and enhanced susceptibility to antimicrobials.

**Table 3:** MIC (in μg/mL) of Peptides in Different *E. coli* Membrane-Modified Strains

	G1-8	G2-1	G2-2	G2-4	G2-10	Pol B	Pol E
MG1655	64	128	**32**	64	64	<2	<2
MG1655wbbl^+^	64	128	**32**	**32**	64	<2	<2
MG1655ΔrfaC	64	128	64	**32**	**32**	<2	<2
MG1655ΔompA	**32**	64	**32**	**32**	**32**	<2	<2
BL21(DE3)ΔompA	64	**32**	**32**	**32**	**32**	4/<2	4
MG1655 MCR-1	64	128	64/32	**32**	64	4	8
MG1655ΔrfaG	64	128	**32**	**32**	64	<2	<2

MG1655ΔompA lacks the major outer-membrane structural porin OmpA, a critical determinant of cell envelope permeability;^33^ the same deletion was examined in the BL21­(DE3) strain, a widely used expression strain (BL21­(DE3)­ΔompA). The strain MG1655 MCR-1 is a colistin-resistant variant that produces a phosphoethanolamine transferase that reduces the affinity of polymyxins for LPS.^34^ MG1655ΔrfaG lacks the enzyme responsible for adding glucose to the LPS core, producing a truncated LPS and increased membrane permeability. The MIC values for the L3 peptide across all constructs were high (>512 μg/mL); therefore, they are not shown here.

Overall, no substantial differences in MIC values were observed among the *E. coli* strains investigated. Peptide **G1-8** showed a one-fold lower MIC against MG1655ΔompA. Peptide **G2-1** showed a one- to two-fold decrease in activity for MG1655ΔompA and BL21­(DE3)­ΔompA. Peptide **G2-2** showed a one-fold increase in activity against MG1655ΔrfaC. Peptide **G2-10** showed a one-fold improvement for MG1655ΔrfaC, MG1655ΔompA, and BL21­(DE3)­ΔompA. Peptide **G2-4** consistently showed a one-fold decrease in MIC across the modified strains. Taken together, the absence of substantial differences in MIC values against these *E. coli* strains across all tested peptides suggests that LPS-dependent membrane integrity plays no major role in the peptide’s mechanism of action.

To further evaluate the peptide’s interaction with the Gram-negative outer membrane, a permeability assay was performed (Figure 5). To this end, outer membrane vesicles (OMVs) from *E. coli* were prepared, containing increased levels of alkaline phosphatase PhoA inside their lumen. OMVs retain the protein and lipid/LPS composition of the bacterial outer membrane they originate from and consequently serve as an *in situ* system to probe the permeability of this membrane.^35,36^ While the enzymatic dephosphorylation of substrates by PhoA encapsulated in intact OMVs is rate-limited by the transmembrane diffusion of the substrate, disruption of this permeability barrier, e.g., by polymyxin antibiotics or detergents like Triton X-100, results in a pronounced increase in the PhoA reaction rate.

**Figure 5:**
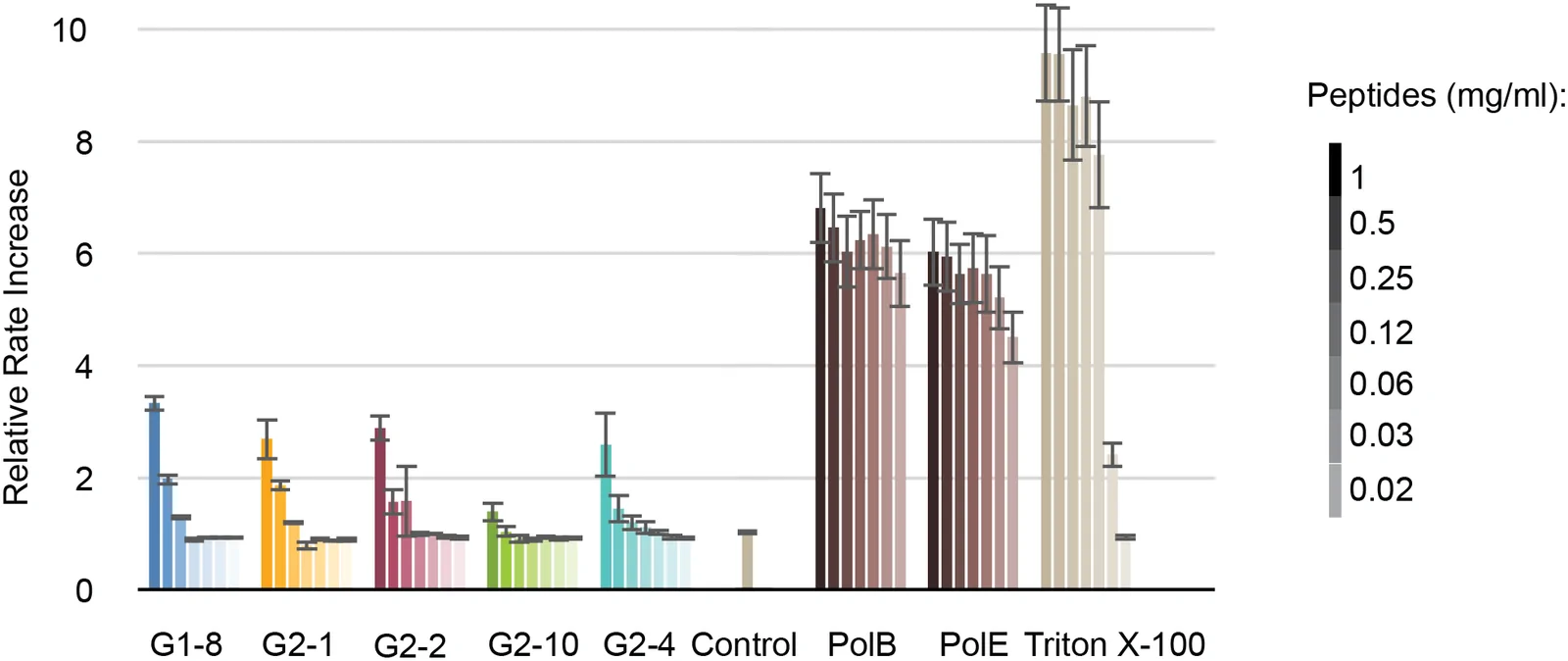
Normalized relative activity of alkaline phosphatase PhoA encapsulated in outer membrane vesicles in the presence of different concentrations of peptides, polymyxin antibiotics (PolB: polymyxin B; PolE: polymyxin E), or Triton X-100 compared to a peptide-free control. Bars represent averages of 3 independent measurements; error bars show standard deviation.

The polymyxin-treated controls showed a substantial increase in membrane permeability at concentrations above their MIC of 1 μg/mL, consistent with membrane disruption as the driving factor in their mechanism of action, similar to OMVs permeabilized with Triton X-100 at concentrations exceeding 62 μg/mL. In contrast, only some of the peptides developed in this study (**G1-8**, **G2-1**, **G2-2**, **G2-4**) resulted in a detectable membrane disruption at high concentrations exceeding 500 μg/mL, well above their MIC values (≈100 μg/mL). In contrast, at lower peptide concentrations, no membrane-lytic activity could be detected. All other peptides did not significantly impact membrane permeability across the tested concentration range.

For peptides **G1-8**, **G2-1**, **G2-2**, and **G2-4**, their membrane-lytic effects correlate with concentration, indicating that the onset of permeabilization occurs at 250–500 μg/mL. Nevertheless, the rate increase observed even at a high concentration of 1 mg/mL is comparable to that of Triton X-100 at 32 μg/mL and substantially lower than the rate increase observed with polymyxins or Triton X-100 at higher concentrations, suggesting that they cause only limited membrane damage.

Despite their structural differences, peptides **G1-8**, **G2-1**, **G2-2**, and **G2-4** showed comparable membrane-lytic activity. However, replacement of Arg with hArg in peptide **G2-10** appears to reduce the lytic activity by a factor of >2, indicating that this modification strongly favors nonlytic behavior. Taken together, and in comparison with the pronounced increase in membrane permeability induced by polymyxins, these results further support the conclusion that lytic activity is not central to the peptide’s mechanism of action.

### DNA Interaction and Disruption of Transcription and Translation

To determine whether the DNA-induced interruption of transcription and translation observed for the lead peptide L3, and the intended dual mode of action, is retained, unchanged, or enhanced, we first proved whenever our designed peptides directly interact with DNA. For this purpose, we performed a fluorescent displacement assay with a standard plasmid DNA-bound fluorophore, DAPI (Figure 6a). At the low DAPI:DNA phosphate ratio used in this assay, DAPI binds preferentially to AT-rich regions in the minor groove of DNA and exhibits strong fluorescence.^37,38^ A decrease in fluorescence upon peptide addition indicated displacement of DNA-bound DAPI. To validate the assay, we used Gly-Gly-Gly (GGG) peptide as a negative control and daunomycin (intercalator and minor-groove binder via its amino sugar)^39^ as a positive control (Figure S41). All five selected peptides (**G1-8**, **G2-1**, **G2-2**, **G2-4**, and **G2-10**) caused concentration-dependent reduction of DAPI-DNA fluorescence, indicating minor groove binding to DNA. This assay does not fully define the peptide-DNA binding geometry, though it provides an evidence that designed AMP peptides directly interact with DNA.

**Figure 6:**
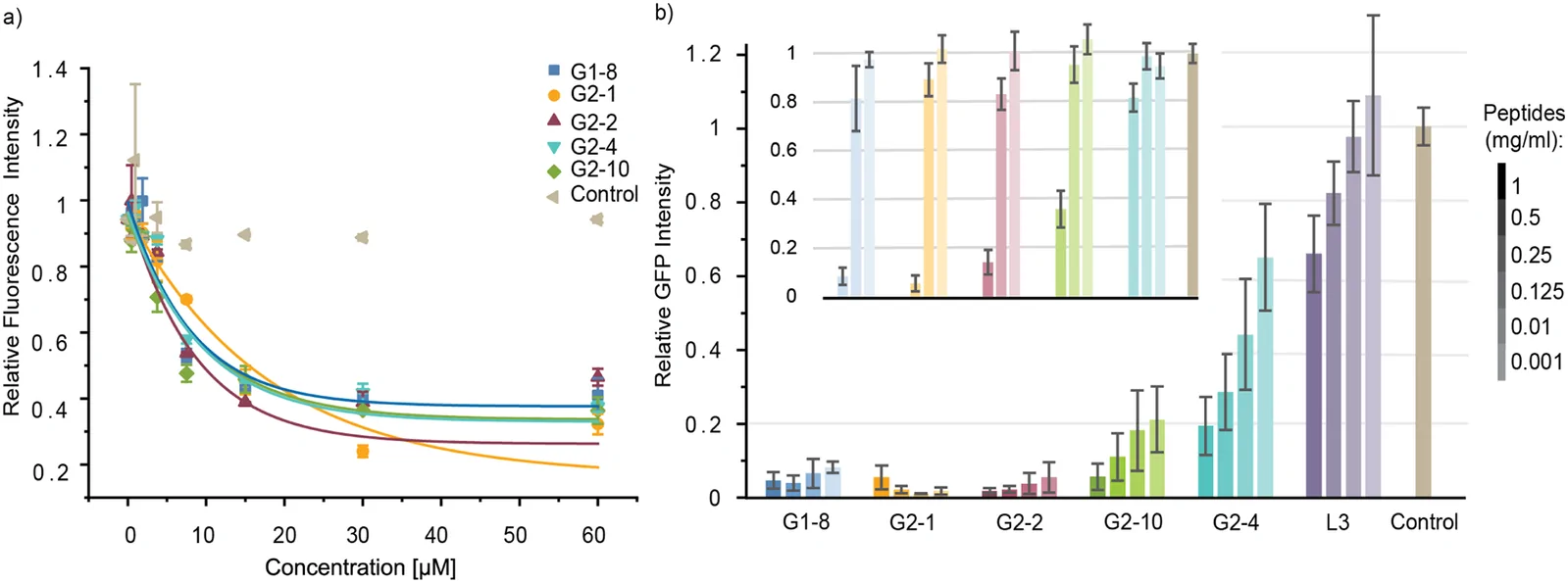
a) Normalized GFP intensity determined in the presence of different concentrations of peptides in an in vitro transcription/translation system. All measurements were normalized to the peptide-free control. Bars represent averages of 3 independent measurements; error bars show standard deviation. In total, 6 different peptide concentrations were measured; in the inset, the lowest 3 concentrations are represented (0.125, 0.01, and 0.001 mg/mL). b) Displacement of DAPI from plasmid DNA. Peak fluorescence (450 nm for DAPI) is normalized to the highest value of fluorescence intensity. Lower fluorescence indicates dye displacement. DAPI to DNA phosphate ratio is 0.025. Peptides (**G1-8**, **G2-1**, **G2-2**, **G2-4**, **G2-10** and negative control peptide GGG) were measured from 60 μM in a 2-fold dilution series. Data points represent average values from two measurements, with standard deviation indicated by the error bar. Lines are exponential fits. Positive assay control is shown in Figure S40.

We further employed an in vitro *E. coli*-based transcription/translation assay to evaluate whether the DNA interaction is connected to inhibition of gene expression independently of cellular metabolism, cell-cycle regulation, and viability, thereby providing a controlled environment for mechanistic evaluation.^40^ We utilized an *E. coli*-based cell extract coupled to the pET expression system, which expresses sfGFP from a plasmid template and is widely regarded as a robust benchmark for measuring IVT efficiency.^41^

In the presence of all five selected peptides (**G1-8**, **G2-1**, **G2-2**, **G2-4**, and **G2-10**), GFP production was markedly reduced compared with both untreated controls, indicating a strong inhibitory effect on transcription and/or translation (Figure 6b). Among the tested peptides, **G1-8**, **G2-1**, and **G2-2** strongly inhibit GFP production at concentrations exceeding 0.1 mg/mL, whereas **G2-10** and **G2-4** show more concentration-dependent inhibition, with **G2-4** showing the strongest concentration dependence and the weakest inhibition. For all peptides, the concentration range at which GFP production is inhibited correlates with their MIC, suggesting that DNA interaction and the consequent disruption of transcription and translation constitute their primary mode of action.

Notably, peptides **G1-8**, **G2-1**, and **G2-2** differ from peptides **G2-10** and **G2-4** by containing hArg instead of Arg and, in the case of peptide **G2-4**, an Ile substitution at position 3 in place of Val. The pronounced functional difference suggests that the Val to Ile substitution exerts the strongest influence on the observed transcriptional and translation inhibitory activity.

Together with the DAPI displacement and OMV permeability assays, these results support DNA interaction and a consequent disruption of DNA-dependent transcription/translation as a major nonlytic part of the antibacterial mechanism.

### Cytotoxicity

Cytotoxicity in human embryonic kidney (HEK293T) and hepatoblastoma (HepG2) cell lines was assessed by measuring metabolic activity 24 h after peptide exposure using a resazurin assay (Figure 7). Cells cultured under identical conditions without treatment served as a negative control and were defined as being 100% viable. None of the peptides exhibited cytotoxic effects, with cell viability exceeding the cytotoxicity threshold of 70% relative to that of the negative control.

**Figure 7:**
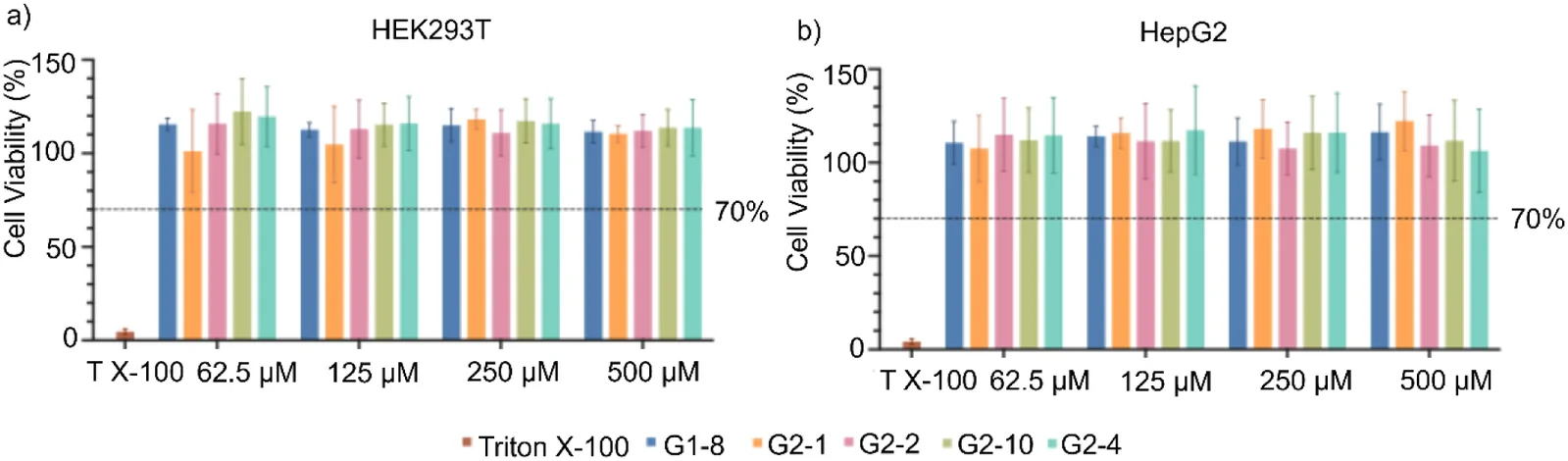
Cell viability 24 h after peptide exposure assessed by a resazurin assay. HEK293T a) and HepG2 b) cell lines were treated with four different peptide concentrations and Triton X-100 as a positive control for cytotoxicity. Data are presented as mean ± SD of three biological replicates for each cell line, where nontreated cells were used for comparison and considered 100% viable. The dotted line indicates the cytotoxicity threshold at 70% cell viability.

## Conclusion

In this study, we systematically generated and evaluated a focused library of 20 peptides derived from the initial lead peptide **L3**, resulting in substantial improvements in antibacterial activity. Several peptides demonstrated markedly enhanced potency, reducing MIC values from >512 μg/mL in the starting sequence to as low as 32 μg/mL for **G2-4**, with additional potent candidates including **G1-8**, **G2-1**, **G2-2**, and **G2-10**, achieving MIC values of 64 μg/mL. Comparative susceptibility testing across resistant and susceptible *E. coli* and *E. marmotae* strains revealed species-specific differences in how antibiotic resistance influenced peptide activity. Whereas *E. coli* showed uniform susceptibility regardless of its extensive resistance profile, *E. marmotae* strains generally showed a more resistant phenotype. These contrasting outcomes likely reflect differences in membrane physiology and the cellular consequences of resistance evolution.

To further elucidate the peptides’ mechanism of action, we assessed their effect on *E. coli* strains with defined membrane alterations, indicating that LPS-dependent membrane integrity is unlikely to be a key determinant. Subsequently, determining the peptides’ membrane-permeabilizing potential using OMVs as an in situ membrane model further supports this conclusion. While polymyxin controls and Triton X-100 produced clear, concentration-dependent increases in membrane permeability, most peptides showed only minimal membrane-lytic activity, even at concentrations far exceeding their MICs. These results indicate that, for peptides in this study, as well as previously shown for the parent peptide **L3**, membrane disruption is limited and is not the primary mechanism of antibacterial action. Using an *E. coli*-based in vitro transcription/translation (IVT) system, we found that all five selected peptides significantly inhibited GFP expression. Peptides **G1-8**, **G2-1**, and **G2-2** showed potent inhibition at concentrations above their MIC, while **G2-10** and **G2-4** had slightly weaker, concentration-dependent effects. No cytotoxicity effects were observed for the five peptides when evaluated in HEK293T and HepG2 cell lines. Together, these data suggest that minor sequence modifications profoundly influence engagement with the transcription/translation machinery. Overall, this work demonstrates that strategic sequence refinement can substantially improve peptide potency while preserving or enhancing their capacity to interfere with essential intracellular processes. These results provide a focused basis for future mechanism-of-action studies for the determination of how the most potent AMPs identified in this study enter the bacterial cell, affect membrane physiology, and translate intracellular target engagement into growth inhibition and bacterial killing. Future hit-to-lead optimization will address pharmacological properties not systematically investigated in this study, including proteolytic stability, while retaining the sequence features responsible for the improved antibacterial potency and nonprimarily lytic mechanism of designed peptides.

## Experimental Section

### Peptide Synthesis, Purification, and Characterization

Peptides were synthesized by automated Fmoc-based SPPS on an INTAVIS MultiPep synthesizer using HBTU and NMM, as reported by Beyer et al.^15^ In short, peptides were synthesized on a Rink amide resin. Global deprotection and cleavage were performed in a mixture of 92.5% TFA, 5% water, and 2.5% TIPS. Peptides G2-8 and G2-9 were subsequently benzylated by incubation with benzoyl chloride and triethylamine (10 equiv relative to loading capacity) in DMF for 2 h at room temperature. Crude and purified peptides were analyzed by analytical RP-HPLC on a Waters e2695 Alliance system (Waters, Milford, MA, USA) employing a Waters 2998 photodiode array (PDA) detector equipped with an ISAspher Xela 100–1.7 C18 column (50 × 2.1 mm). HPLC eluent A was water (0.1% trifluoroacetic acid (TFA)), and eluent B was acetonitrile (0.1% TFA) (detection at 214 nm). Preparative-scale purification of the peptides was achieved by employing a Waters 1525 binary gradient pump and a Waters 2998 PDA detector with HPLC eluent A (water, 0.1% TFA) and eluent B (acetonitrile, 0.1% TFA). The molecular weight of the purified compounds was confirmed by ESI mass spectrometry on a Waters SYNAPT G2-Si HD-MS spectrometer equipped with a Waters Acquity UPLC system (Waters, Milford, MA, USA). Leu-enkephalin was used as a reference compound for the high-resolution measurements. Chromatograms are reported in the SI (Table S1, Figures S1–S42). The purity of peptides was >95% based on HPLC analysis.

### Strains and Growth Conditions for MIC Testing

*E. coli*, *E. marmotae*, *S. aureus*, and *K. pneumoniae* strains were cultured aerobically on LB agar plates and incubated at 37 °C. *C. albicans* was cultivated on bactoyeast-bactopeptone agar plates at 30 °C.

### Minimal Inhibitory Concentration (MIC)

MIC assays for bacteria were performed according to the Clinical and Laboratory Standards Institute (CLSI) guidelines, with minor modifications. Serial 2-fold dilutions of each peptide were prepared in cation-adjusted Mueller–Hinton broth (MHB) in sterile 96-well microtiter plates and inoculated with *E. coli* (CCUG 17620), *E. marmotae*, *S. aureus* (CCUG 1800T), or *K. pneumoniae* (CCUG 28450) at a final density of 2–7 × 10^5^ CFU/mL. Plates were incubated aerobically at 37 °C for 16–21 h before MIC determination.

The yeast MIC assay was conducted following the procedures described by Mesa et al.^42^ and the recommendations of the Subcommittee on Antifungal Susceptibility Testing of the ESCMID European Committee for Antimicrobial Susceptibility Testing,^43^ with minor modifications. Serial 2-fold peptide dilutions were prepared in a medium consisting of 30% bactoyeast/bactopeptone/glucose (1:2:2) and 70% cation-adjusted MHB in sterile 96-well plates, followed by inoculation with *Candida albicans* CCUG 31028 at a final density of 2–7 × 10^5^ CFU/mL. Plates were incubated at 30 °C for 20 h before MIC evaluation.

### CD Spectroscopy

Circular dichroism spectra were recorded on a Chirascan Applied Photophysics in a cuvette with a length of 1 cm between 180 and 280 nm and a bandwidth of 1 nm with a step size of 1 nm and a response time of 1 s per point. CD spectra were recorded as absorbance in mdeg at room temperature. The background was measured and subtracted from the CD spectra from the samples by measuring a blank sample with the corresponding solvent. Measurements were run as triplicates and averaged. Peptides were measured in water and TFE at 50 μM. Deconvolution was performed using Beta Structure Selection (BeStSel), a freely accessible web server (BeStSel - Protein Circular Dichroism Spectra Analysis).^44^

### NMR

Solution NMR experiments were performed at 280 K on a Bruker Avance III HD 900 MHz spectrometer. The peptide sample was dissolved in 90% H_2_O/10% D_2_O from a freeze-dried solid. Data were acquired and processed with Topspin 5.0.0 (Bruker, Rheinstetten, Germany) and analyzed with CCPN.^45^ Automated chemical shift assignments and distance restraints were performed and assessed using ARTINA.^46^ The proton resonance assignment was performed using a combination of 2D [1H,1H]-TOCSY (80 ms spinlock time) and [1H,1H]-NOESY experiments. Distance constraints were extracted from [1H,1H]-NOESY spectra acquired with a 200 ms mixing time. Structure calculations were performed with YASARA structure (Supporting Information, Table S3).^47–49^ Structures were refined in water at pH 3.

### Fluorescence Displacement Assay

The DNA (sfGFP) concentration in complexes with DAPI (Sigma-Aldrich) was at 4 μM of phosphates. The DNA-DAPI complex was at a dye/DNA phosphate ratio of 0.025, at which the minor groove binding mode with strong fluorescence dominates.^37^ Peptides and daunomycin were dissolved in Milli-Q ultrapure water to a stock concentration. All further solutions were prepared in 25 mM Tris, 5 mM NaCl buffer, pH 7.4. Fluorescence was excited at 355 nm, and the emission wavelength was 450 nm. The emission was recorded using a microplate reader (Tecan Infinite 200 PRO) in a black 384-well plate. Each well was loaded with 90 μL of DAPI/DNA with a final concentration of 0.1 μM DAPI and 4 μM DNA phosphates and 10 μL of peptide solution. Peptides were measured in a 2-fold dilution series at 60, 30, 15, 7.5, 3.8, 1.9, 0.9, 0.5, and 0 μM. Daunomycin was measured in a 2-fold dilution series starting with 2 μM. Readings were recorded in duplicate, and their standard deviation was calculated. Fluorescence was recorded 5 to 10 min after adding samples to the wells, when no further changes in the spectrum were observed. The negative control peptide GGG was purchased from Bachem.

### In Vitro Transcription/Translation Assay

Crude S12 extracts for cell-free protein synthesis were prepared as described by Kwon et al.^41^ Briefly, *E. coli* strain BL21­(DE3)* was grown to OD_600_ ≈ 3.0 in 2xYPTG medium at 37 °C following induction with 1 mM IPTG at OD_600_ ≈ 0.6 for the T7 RNA polymerase-based assay and without induction for the endogenous RNA polymerase-based assay. Extracts were prepared by lysing cells using sonication following resuspension at a ratio of 1:1.25 (w/v) in extraction buffer (10 mM Tris-acetate, pH 8.2, 14 mM magnesium acetate, 60 mM potassium acetate, 1 mM DTT, 1x Roche Complete EDTA-free). Lysates were cleared by centrifugation (12,000 × *g* for 10 min). For the assay, peptides (as well as a peptide-free control) were diluted to final concentrations of 1, 0.5, 0.25, and 0.125 mg/mL, or 1, 0.1, 0.01, and 0.001 mg/mL, respectively, in a 50 μL total reaction volume containing final concentrations of the respective cell extract 31% (v/v), Mg­(OAc)_2_ 14 mM; ammonium hydroxide 27.4 mM; d-glutamic acid 212 mM; Hepes-KOH pH 6.5 52.5 mM; KOH 230 mM; ATP 1.1 mM; GTP 0.8 mM; CTP 0.8 mM; UTP 0.8 mM; cAMP 0.64 mM; folinic acid 68 μM; DTT 1.7 mM; creatine phosphate 63 mM; malic acid 4.4 mM; succinic acid 1.5 mM; tRNA 0.175 mg/mL; Protector RNase inhib. 8 U/mL; Roche Complete EDTA-free 1x; proteinogenic amino acids (1 mM each); serine 2 mM; glutamine 4 mM; creatine kinase 125 μg/mL;^50,51^ as well as DNA template (sfGFP in pCPR-0012).^52,53^ Following preparation at 4 °C, reactions were started by shifting the temperature to 30 °C, followed by incubation for 2 h. GFP fluorescence was monitored using a microplate reader (Tecan Infinite 200 PRO) using an excitation wavelength of 475 nm and an emission wavelength of 515 nm, and endpoint values were used for analysis. All measured values were normalized to the peptide-free control.

### Membrane Perforation Assay

OMVs carrying active alkaline phosphatase inside their lumen were prepared following the method previously described.^54^ Briefly, *E. coli* strain BL21­(DE3)­ΔompA^55^ carrying plasmid pY321 was grown at 37 °C in LB medium supplemented with 10 μM ZnCl_2_ and 50 μg/mL kanamycin. When cultures reached an optical density (600 nm) of 0.4, protein production was induced by the addition of 50 μM IPTG, and growth was maintained for 2 h and 30 min. Cells were removed by centrifugation at 10,000 × *g* for 10 min, and cleared supernatants were filtered using 0.45 μm bottle-top filters. OMVs were collected by centrifugation at 38,400 × *g* for 2 h, washed in buffer (20 mM Tris pH 8, 150 mM NaCl, 1 mM CaCl_2_, 0.5 mM MgCl_2_, and 10 μM ZnSO_4_), filtered using 0.45 μm syringe filters, recollected by centrifugation at 74,500 × *g* for 45 min, and resuspended in buffer. The total protein concentration was determined spectroscopically by correcting the spectra for OMV light scattering and assuming 1 Abs = 1 mg/mL, as described.^54^

The activity of arctic phosphatase encapsulated in OMVs was determined by monitoring the dephosphorylation of 4-methylumbelliferyl phosphate in clear flat-bottom half-area 96-well microplates (Corning) by measuring the increase in fluorescence intensity (λ_EX_ = 360 nm, λ_EM_ = 449 nm) on an Infinite 200 PRO microplate reader (Tecan) for 30 min. 40 μL of OMV suspension at a total protein concentration of 7.5 μg/mL was incubated with 10 μL of peptide solution (concentrations as indicated) for 5 min before the addition of 50 μL of 4-methylumbelliferyl phosphate solution (50 μM in buffer). The relative phosphatase activity was determined based on the reaction rate of the first 10 min of the reaction as (∂FI/∂t)­peptide/(∂FI/∂t)­buffer. For each condition, three independent replicates were measured.

### Cytotoxicity

The cytotoxicity of the peptides in human embryonic kidney (HEK293T) and hepatoblastoma (HepG2) cell lines was evaluated using a resazurin-based assay. Cells were seeded at a density of 10,000 cells per well in 96-well plates and cultured for 24 h prior to peptide treatment at final concentrations of 62.5 (91–94 μg/mL), 125 (183–188 μg/mL), 250 (366–377 μg/mL), and 500 μM (731–754 μg/mL). Triton X-100 (0.1% v/v) was used as a positive control for cytotoxicity. Twenty-four hours after treatment, cell viability was assessed by adding resazurin to a final concentration of 0.015 mg/mL, followed by 4 h incubation and fluorescence intensity quantification. Measurements were performed using a Hidex Sense microplate reader with an excitation wavelength of 544 nm and an emission wavelength of 590 nm.

## Supplementary Material





## Notes

Experimental details, materials, methods, and characterization information (PDF)
Molecular formula strings for all newly synthesized compounds (CSV)

## Abbreviations

AMP: antimicrobial peptide
AMR: antimicrobial resistance
ATP: adenosine triphosphate
*C. albicans*: *Candida albicans*
cAMP: cyclic adenosine monophosphate
Cit: citrulline
CTP: cytidine triphosphate
DAPI: 4′,6-diamidino-2-phenylindole
DTT: dithiothreitol
*E. coli*: *Escherichia coli*
ETEC: enterotoxigenic *E. coli*
ExPEC: extraintestinal *E. coli*
hArg: homoarginine
*HBTU*: hexafluorophosphate benzotriazole tetramethyl uronium
Hep: hepatoblastoma
Hyp: hydroxyproline
IPTG: isopropyl β-D-1-thiogalactopyranoside
IVT: in vitro transcription/translation
*K. pneumoniae*: *Klebsiella pneumoniae*
LB: lysogeny broth
MHB: Mueller–Hinton broth
Nle: norleucine
*NMM*: *N*-Methylmorpholine
OMVs: outer membrane vesicles
Orn: ornithine
pET: plasmid for *E. coli*
PolB: polymyxin B
PolE: polymyxin E
*S. aureus*: *Staphylococcus aureus*
sfGFP: superfolded GFP
ST: multi-locus sequence type
TFE: 2,2,2-trifluoroethanol
Tyr­(NO_2_): 3-nitro-Tyr
UPEC: uropathogenic *E. coli*
UTP: uridine triphosphate
YPTG: peptone-tryptone-yeast extract-glucose medium
