# Supplementary material for: Dual-Mechanism Antimicrobial Peptides from a Nature-Inspired Scaffold
Source: J Med Chem. 2026 Jul 24;69(15):18580–91. doi: 10.1021/acs.jmedchem.6c00952 (PMC13492378; doi:10.1021/acs.jmedchem.6c00952)
Supplement: Supplementary file 1 [file jm6c00952_si_001.pdf]

Dual-Mechanism Antimicrobial Peptides from a Nature-Inspired Scaffold

Luisa I. Beyer, a,b,c Johannes Thoma, a,c Silvana Lord Smits, a,c Annie Justh de Neczpal, a,c Hanna Mårtensson, c,d Julia Leandersson, a Maya Tabbaa, a Anne Farewell, a,b Åsa Sjöling, a,b,, Alexandra Stubelius, d Alesia A. Tietze a,b,c*

a University of Gothenburg, Department of Chemistry and Molecular Biology, Medicinaregatan 16, 413 90 Gothenburg

b Wallenberg Centre for Molecular and Translational Medicine, 405 30 Gothenburg, Sweden

c Center for Antibiotic Resistance Research in Gothenburg, The University of Gothenburg, Box 100, 405 30 Gothenburg, Sweden

d Chalmers University of Technology, Department of Life Sciences, Kemigården 4, 412 96 Göteborg, Sweden

Corresponding address:

*University of Gothenburg, Department of Chemistry and Molecular Biology, Wallenberg Centre for Molecular and Translational Medicine, Natrium, Medicinaregatan 16, 41390 Gothenburg, Sweden

*Email: alesia.a.tietze@gu.se

Contents

Analytical data peptides S2

CD deconvolution and spectra S16

NMR S17

Fluorescence Displacement Assay DAPI S18

Analytical data peptides

Table S1. LC-MS data

Peptide	Sum formula	Calculated MW* [g/mol]	Experimental MW* [g/mol]	Δppm	Retention time** [min]
**G1-1**	C67H99N17O13	450.9281	450.9266	3.32	3.9
**G1-2**	C67H99N17O13	450.9281	450.9266	3.32	4.1
**G1-3**	C67H99N17O13	450.9281	450.9266	3.32	3.9
**G1-4**	C64H101N17O12	434.2683	434.2629	12.43	4.0
**G1-5**	C63H97N17O13	434.2562	434.2538	5.52	3.5
**G1-6**	C68H99N17O13	454.9281	454.9251	6.59	3.5
**G1-7**	C69H103N17O13	460.2719	460.2714	1.08	3.9
**G1-8**	C69H103N23O13	488.278	488.2764	3.27	4.0
**G2-1**	C69H102N24O15	503.273	503.2749	-3.77	4.2
**G2-2**	C70H105N23O13	492.9499	492.9503	-0.81	4.2
**G2-3**	C67H99N23O14	484.2659	484.2632	5.57	3.8
**G2-4**	C70H105N23O13	492.9499	492.9503	-0.81	4.2
**G2-5**	C69H101N23O13	487.6061	487.6064	-0.61	3.9
**G2-6**	C63H100N22O13	458.6025	458.6048	-5.01	3
**G2-7**	C64H100N24O13	471.9379	471.9391	-2.54	2.8
**G2-8**	C71H104N24O14	506.6133	506.6142	-1.77	4.0
**G2-9**	C70H104N22O14	493.2779	493.2749	6.08	5.2
**G2-10**	C72H109N23O13	502.2937	502.2914	4.57	4.1
**G2-11**	C69H100N20O16	489.262	489.2606	2.86	4.8

* Mass peaks detected as $[M+3H]^{3+}$; except for S1 $[M+1H]^{1+}$ and S2 $[M+2H]^{2+}$

** All spectra were measured with gradient from 10 to 50% acetonitrile in water over 10 min

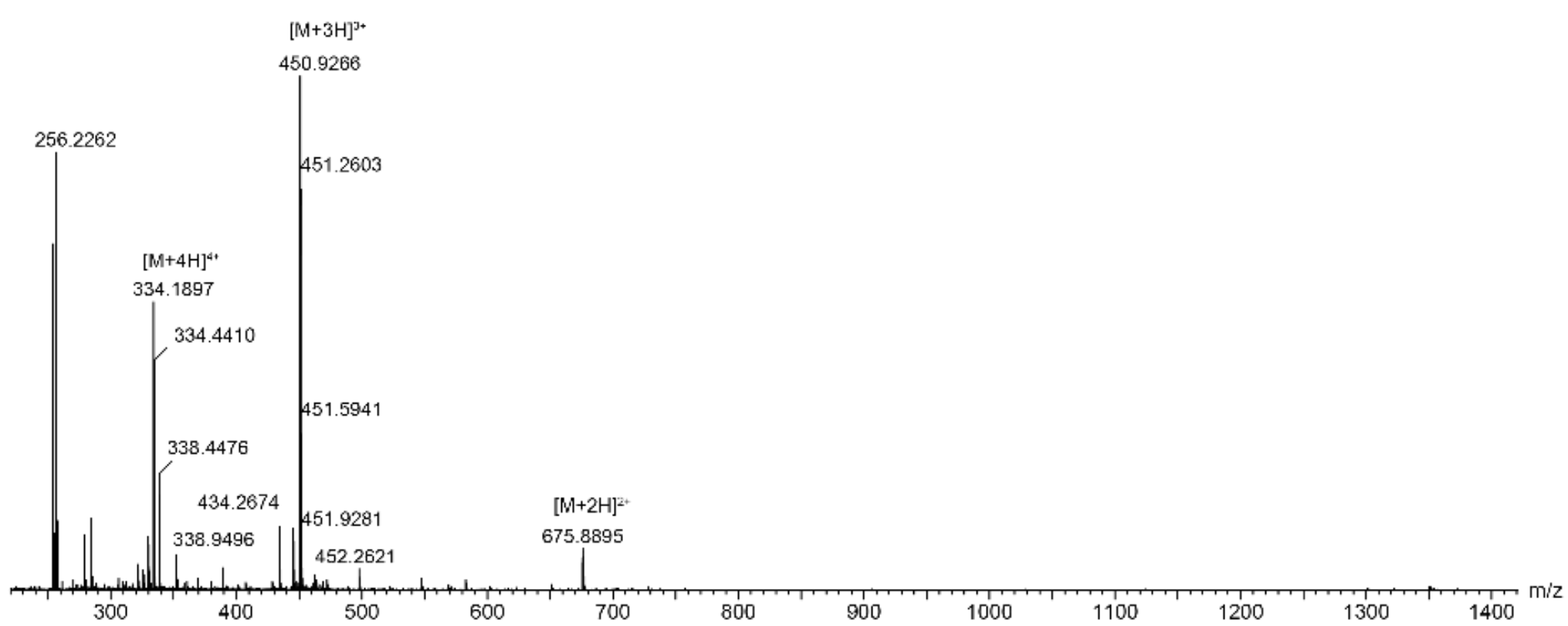

Figure S1: High resolution mass spectrum for peptide G1-1.

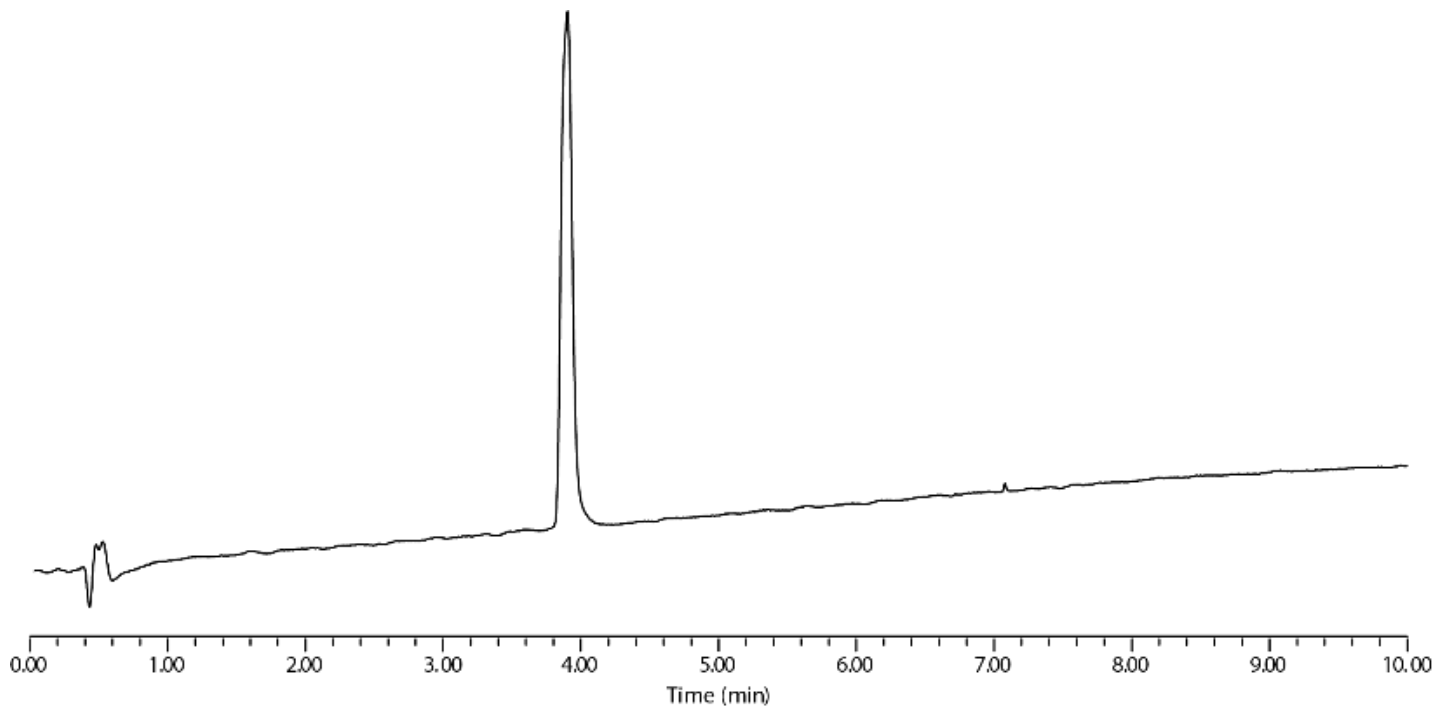

Figure S2: RP-HPLC chromatogram (gradient from 10 to 50% acetonitrile in water over 10 min at 2 ml/min, detection at 214 nm) for peptide G1-1.

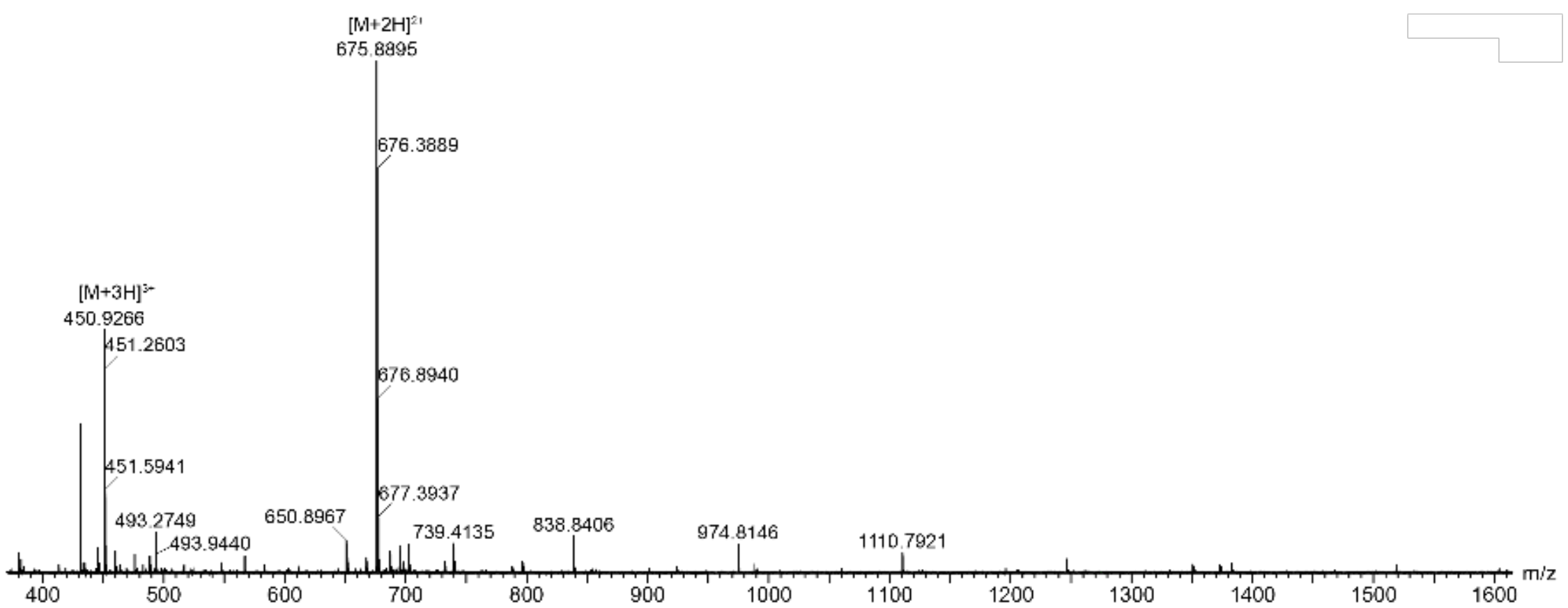

Figure S3: High resolution mass spectrum for peptide G1-2.

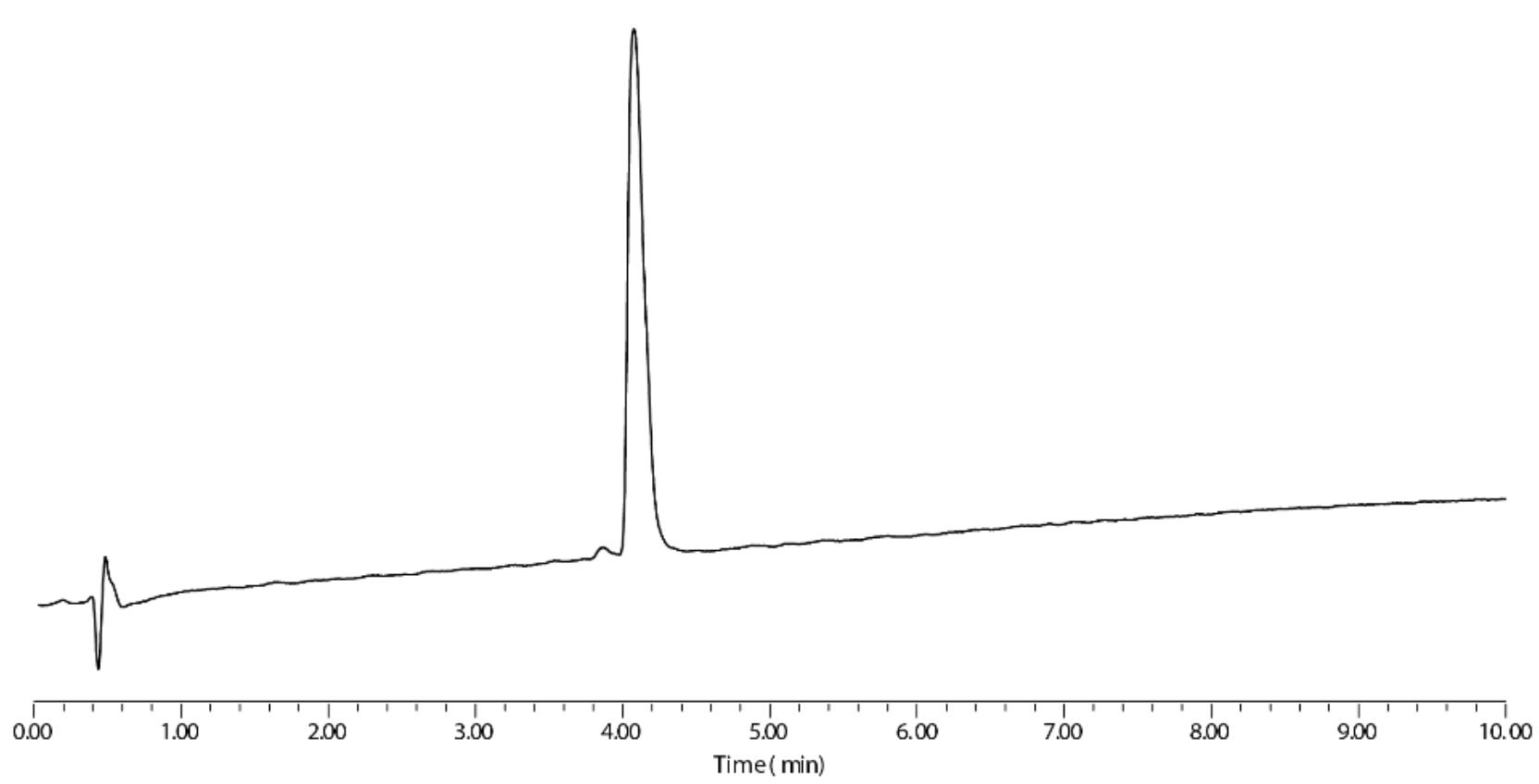

Figure S4: RP-HPLC chromatogram (gradient from 10 to 50% acetonitrile in water over 10 min at 2 ml/min, detection at 214 nm) for peptide G1-2.

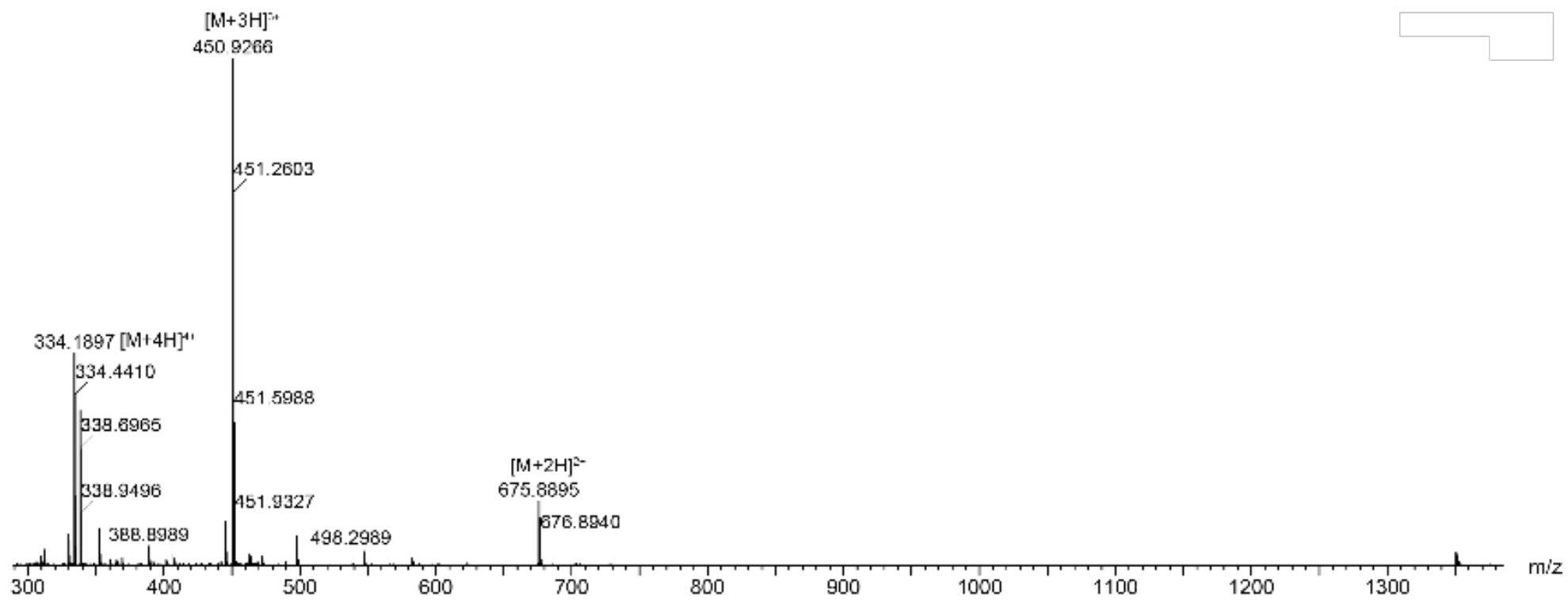

Figure S5: High resolution mass spectrum for peptide G1-3.

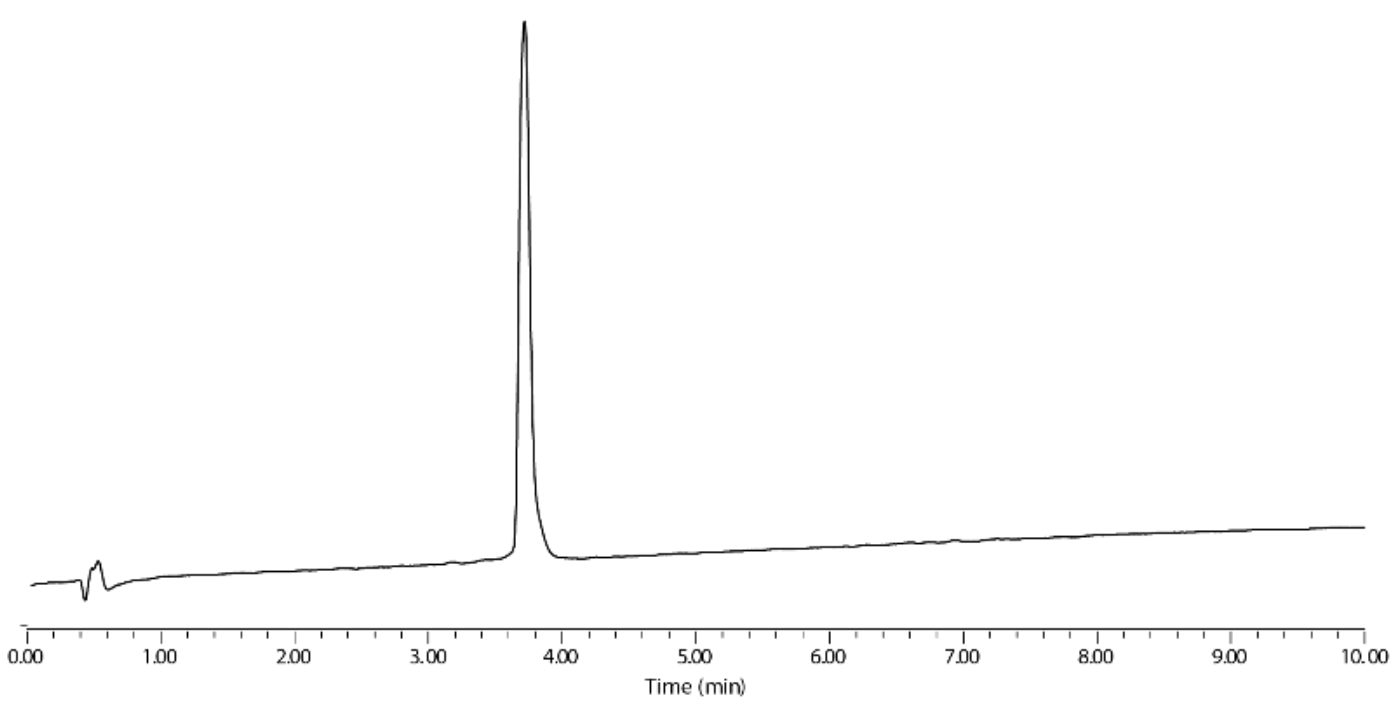

Figure S6: RP-HPLC chromatogram (gradient from 10 to 50% acetonitrile in water over 10 min at 2 ml/min, detection at 214 nm) for peptide G1-3.

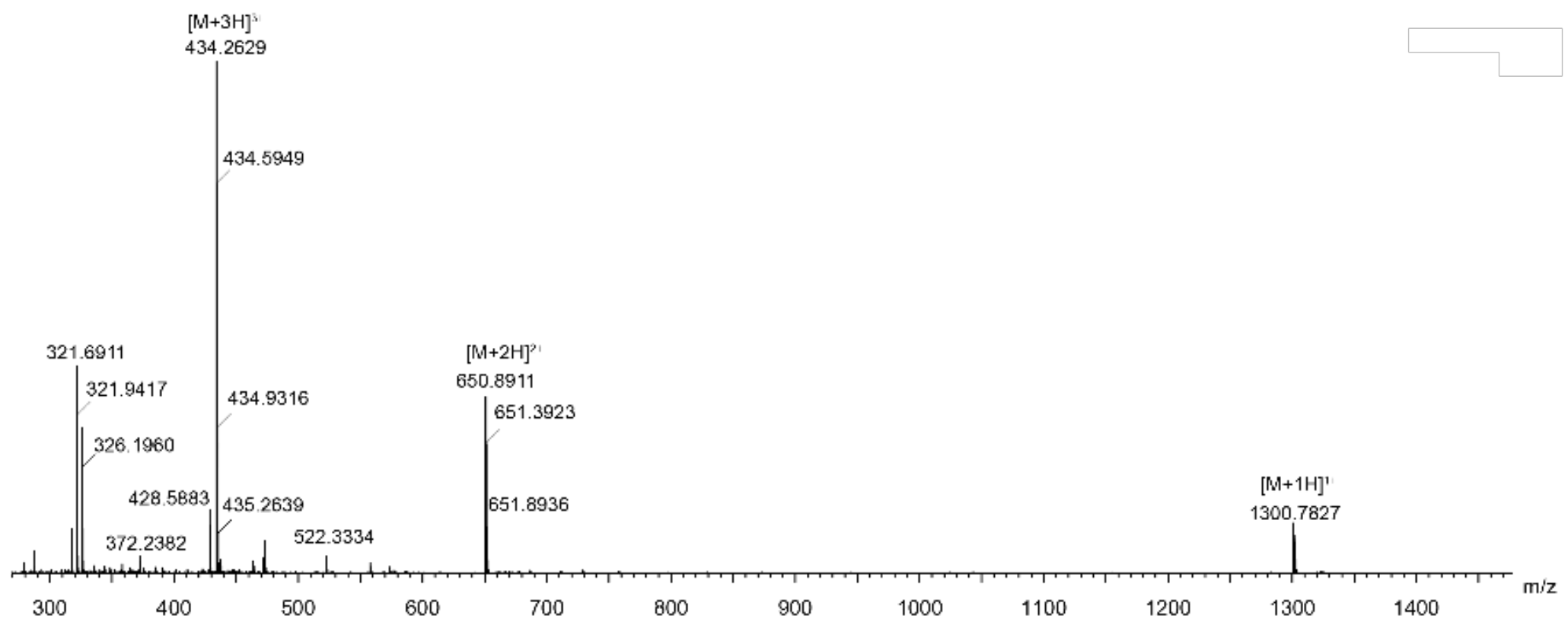

Figure S7: High resolution mass spectrum for peptide G1-4.

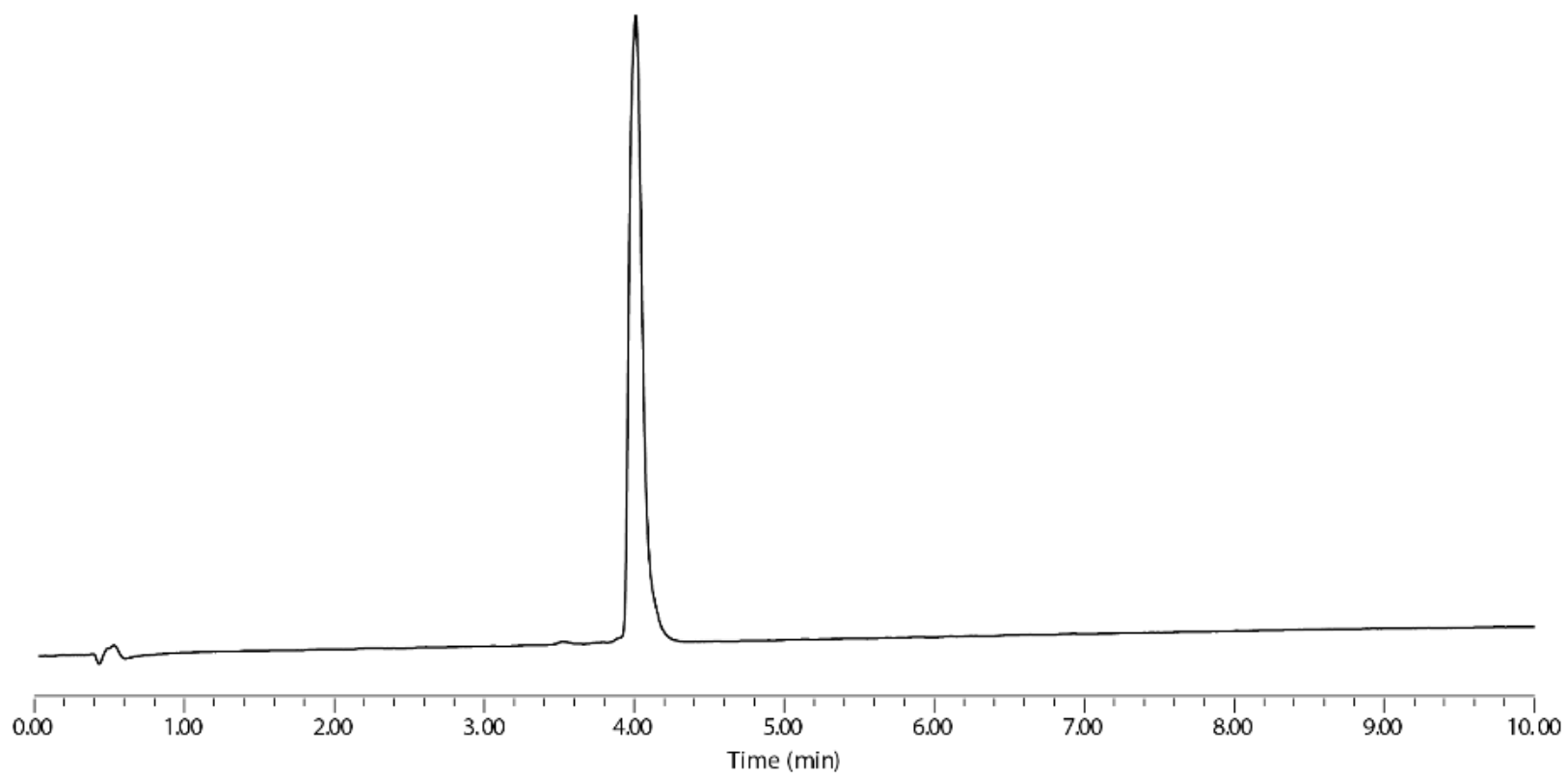

Figure S8: RP-HPLC chromatogram (gradient from 10 to 50% acetonitrile in water over 10 min at 2 ml/min, detection at 214 nm) for peptide G1-4.

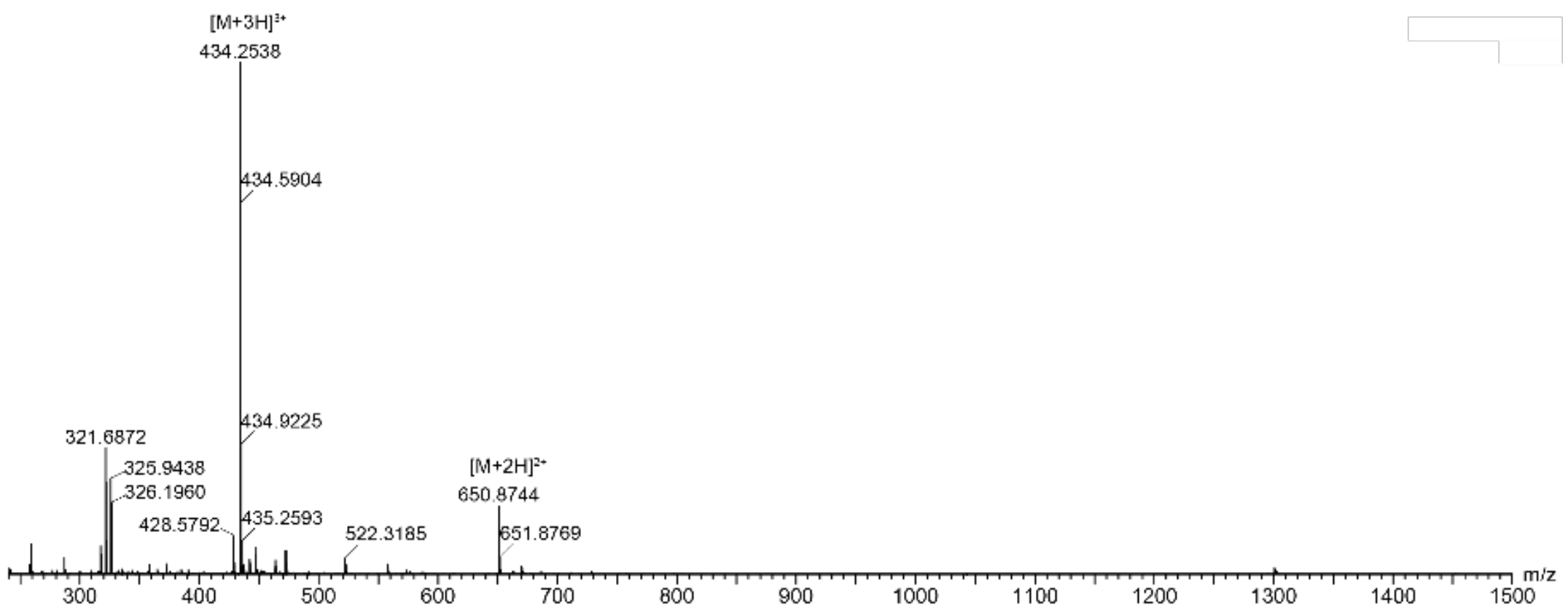

Figure S9: High resolution mass spectrum for peptide G1-5.

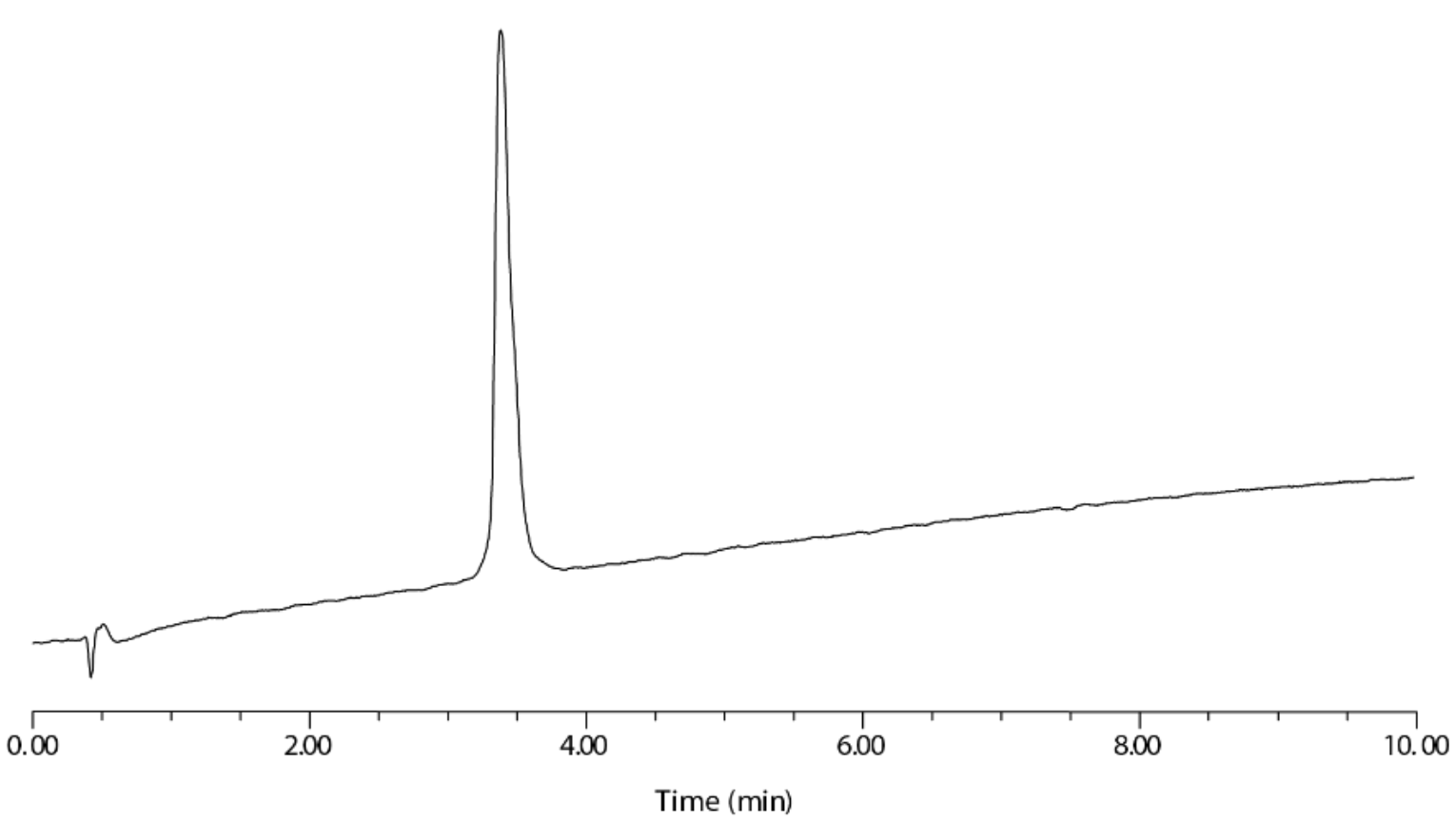

Figure S10: RP-HPLC chromatogram (gradient from 10 to 50% acetonitrile in water over 10 min at 2 ml/min, detection at 214 nm) for peptide G1-5.

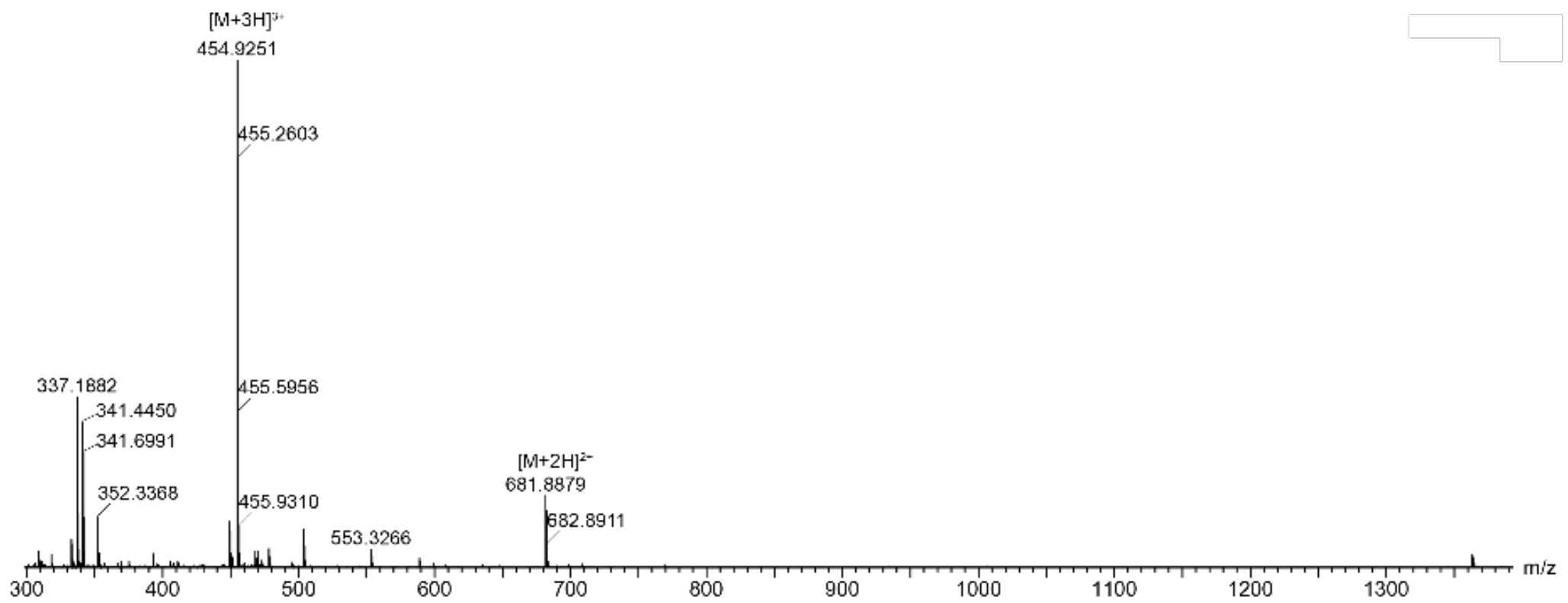

Figure S11: High resolution mass spectrum for peptide G1-6.

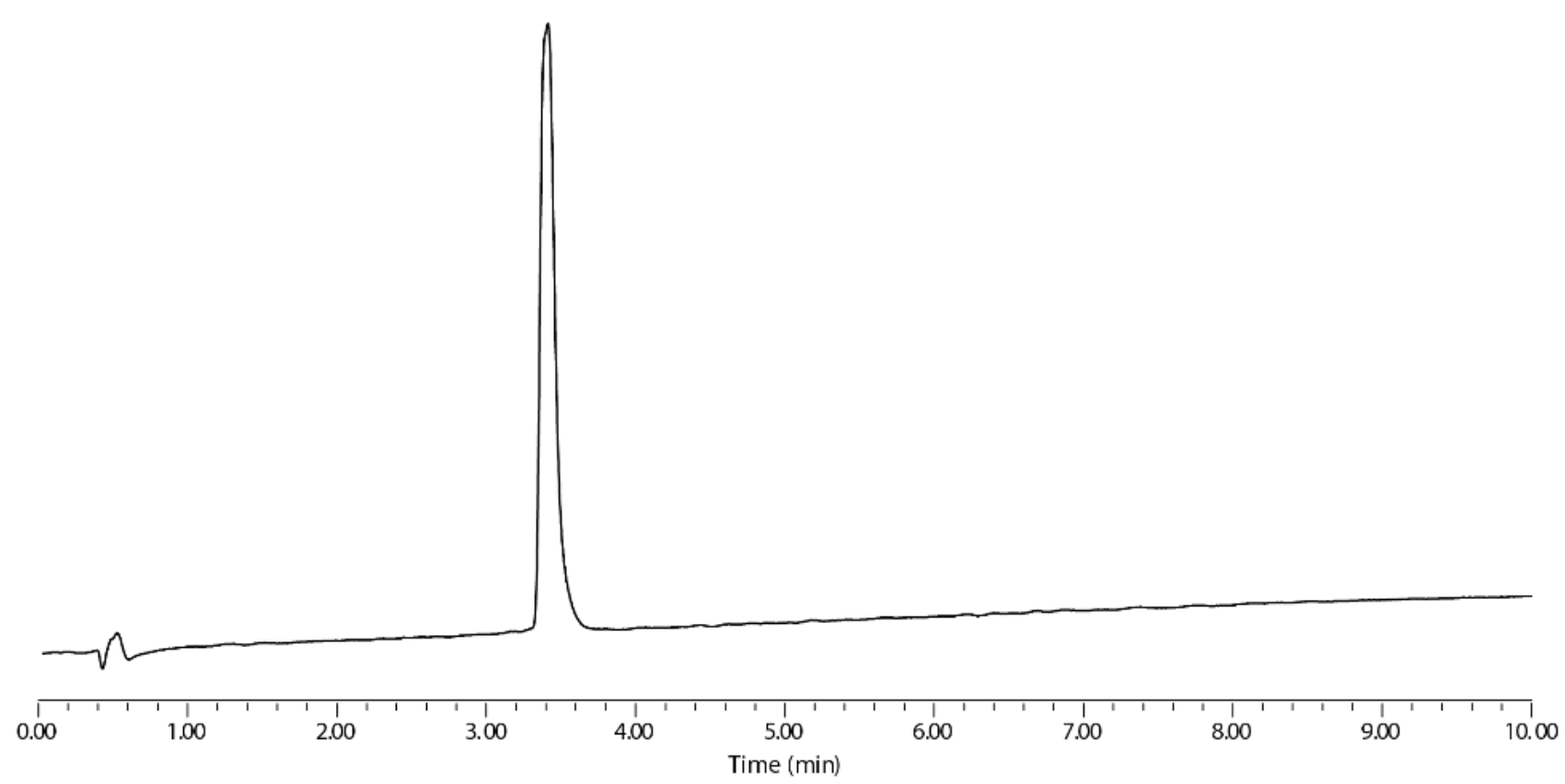

Figure S12: RP-HPLC chromatogram (gradient from 10 to 50% acetonitrile in water over 10 min at 2 ml/min, detection at 214 nm) for peptide G1-6.

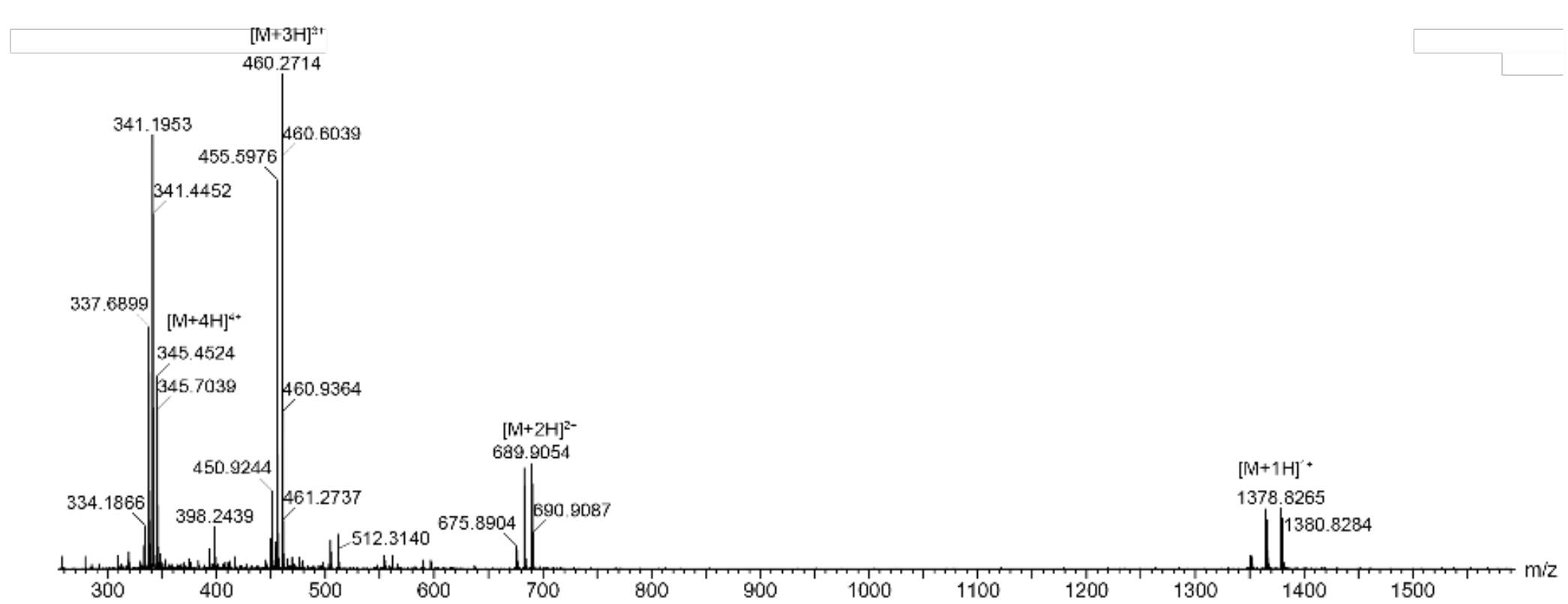

Figure S13: High resolution mass spectrum for peptide G1-7.

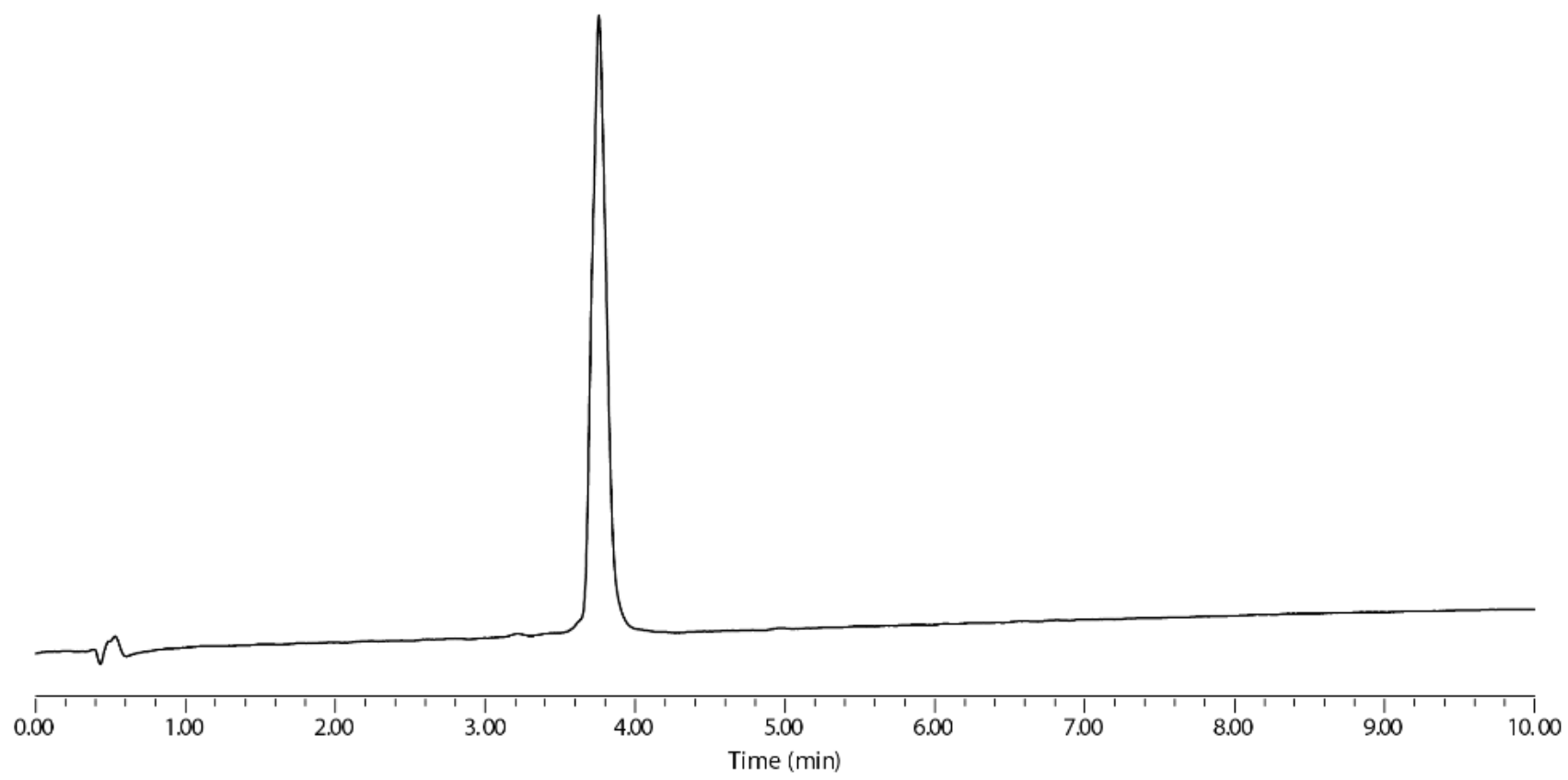

Figure S14: RP-HPLC chromatogram (gradient from 10 to 50% acetonitrile in water over 10 min at 2 ml/min, detection at 214 nm) for peptide G1-7.

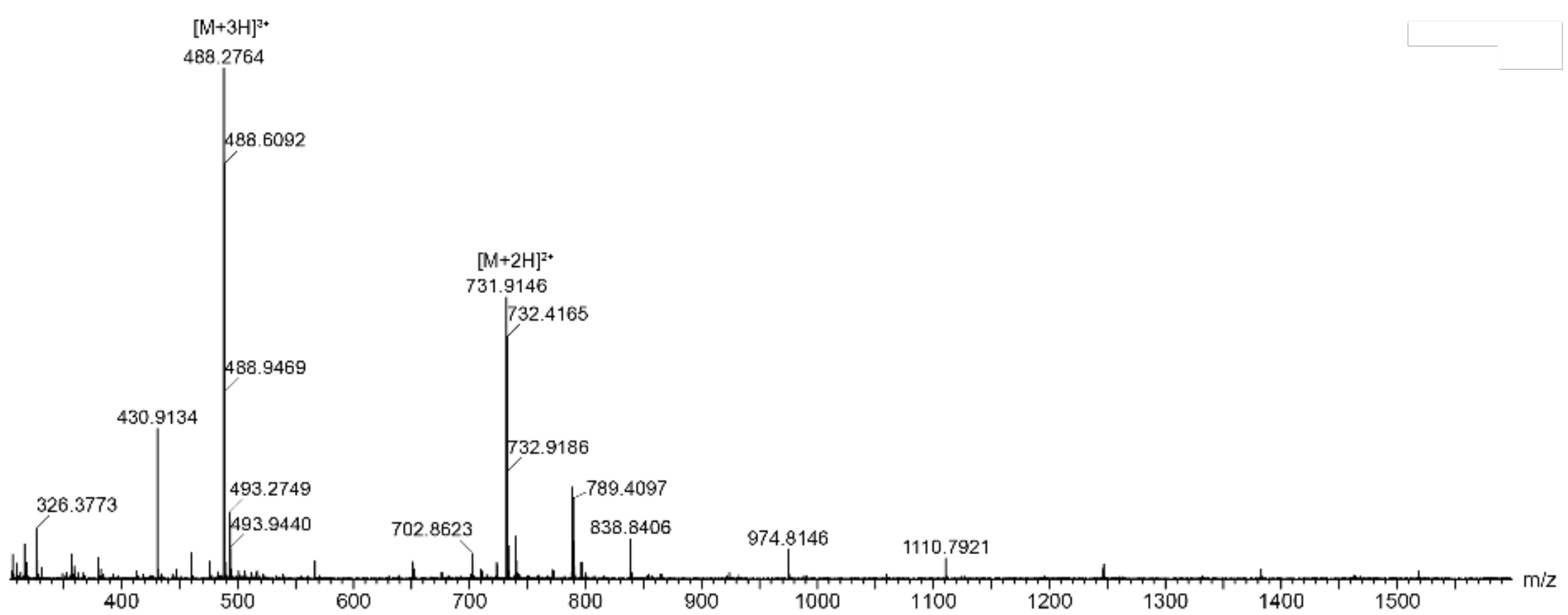

Figure S15: High resolution mass spectrum for peptide G1-8.

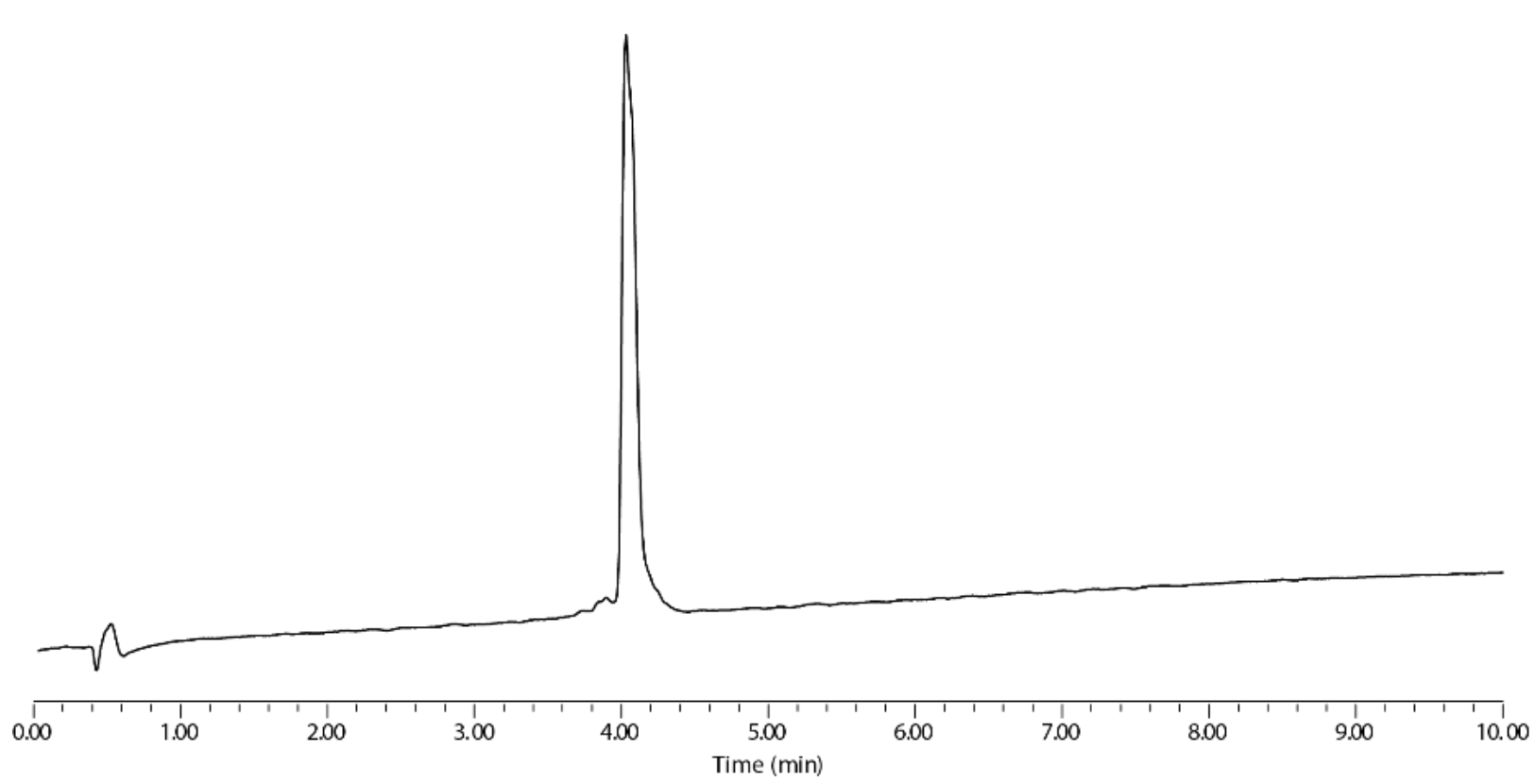

Figure S16: RP-HPLC chromatogram (gradient from 10 to 50% acetonitrile in water over 10 min at 2 ml/min, detection at 214 nm) for peptide G1-8.

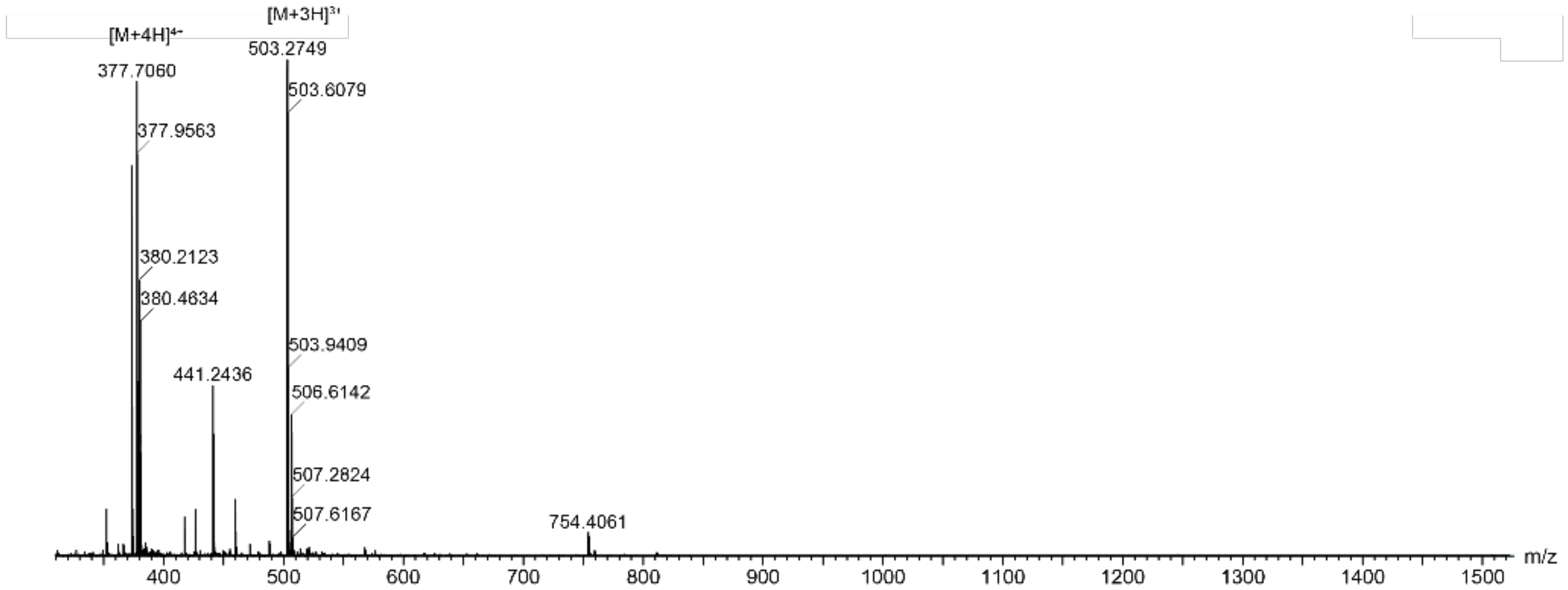

Figure S17: High resolution mass spectrum for peptide G2-1.

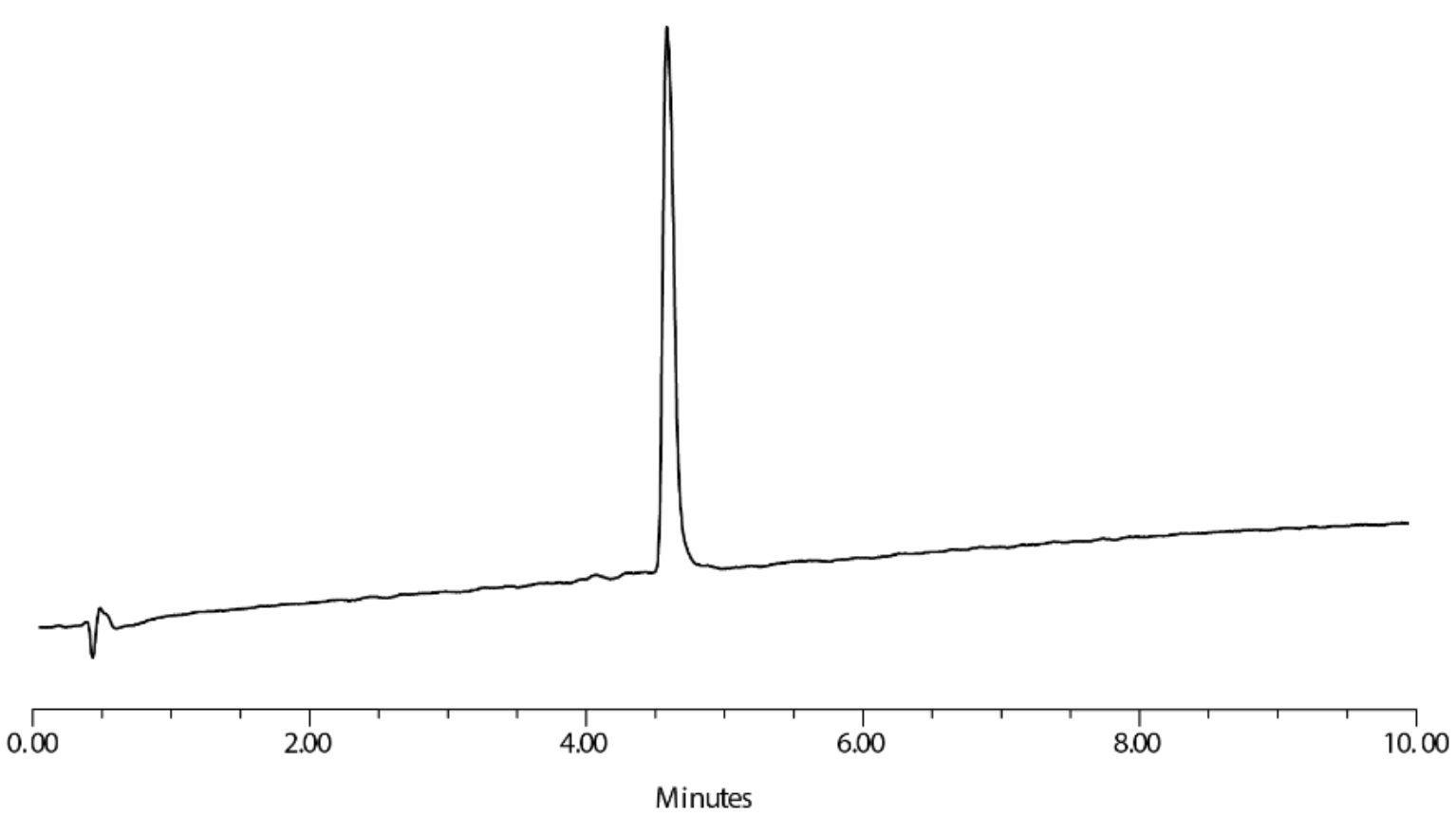

Figure S18: RP-HPLC chromatogram (gradient from 10 to 50% acetonitrile in water over 10 min at 2 ml/min, detection at 214 nm) for peptide G2-1.

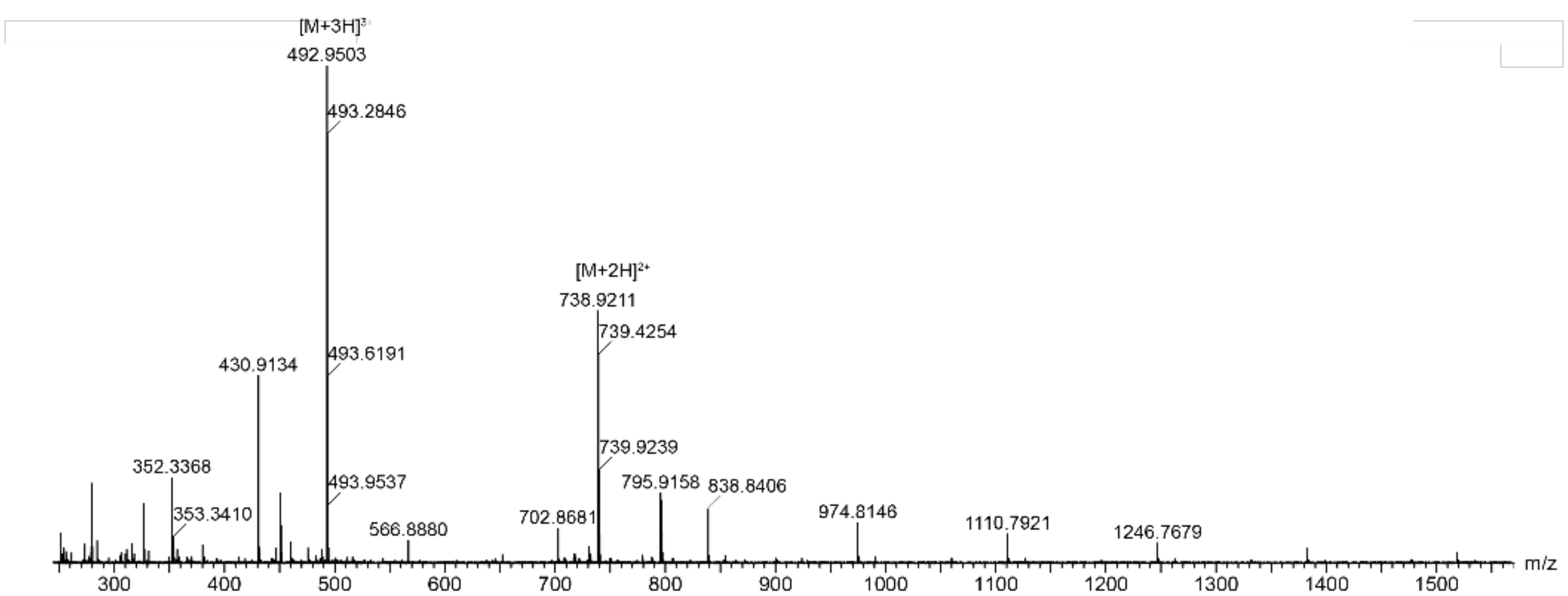

Figure S19: High resolution mass spectrum for peptide G2-2.

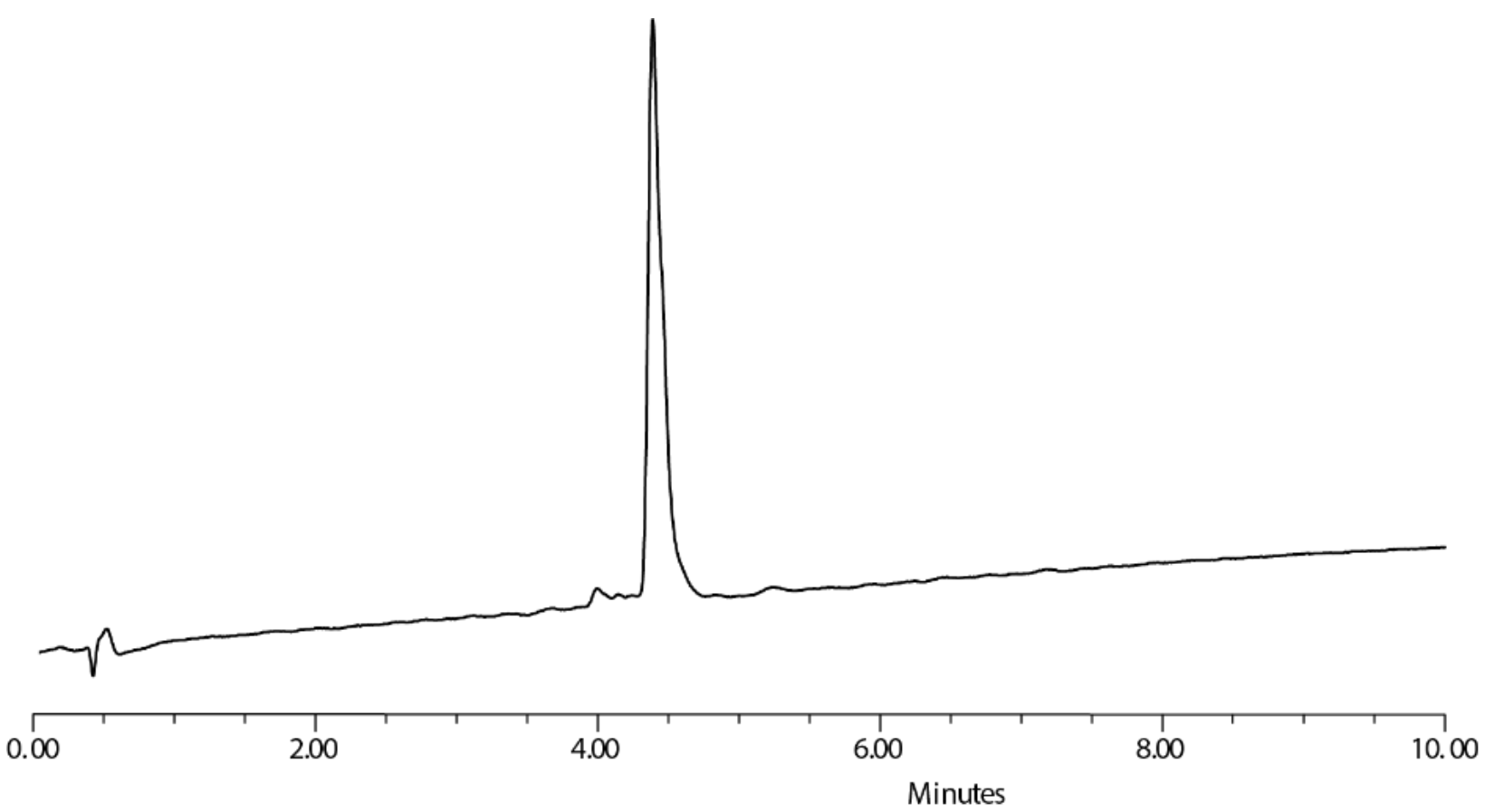

Figure S20: RP-HPLC chromatogram (gradient from 10 to 50% acetonitrile in water over 10 min at 2 ml/min, detection at 214 nm) for peptide G2-2.

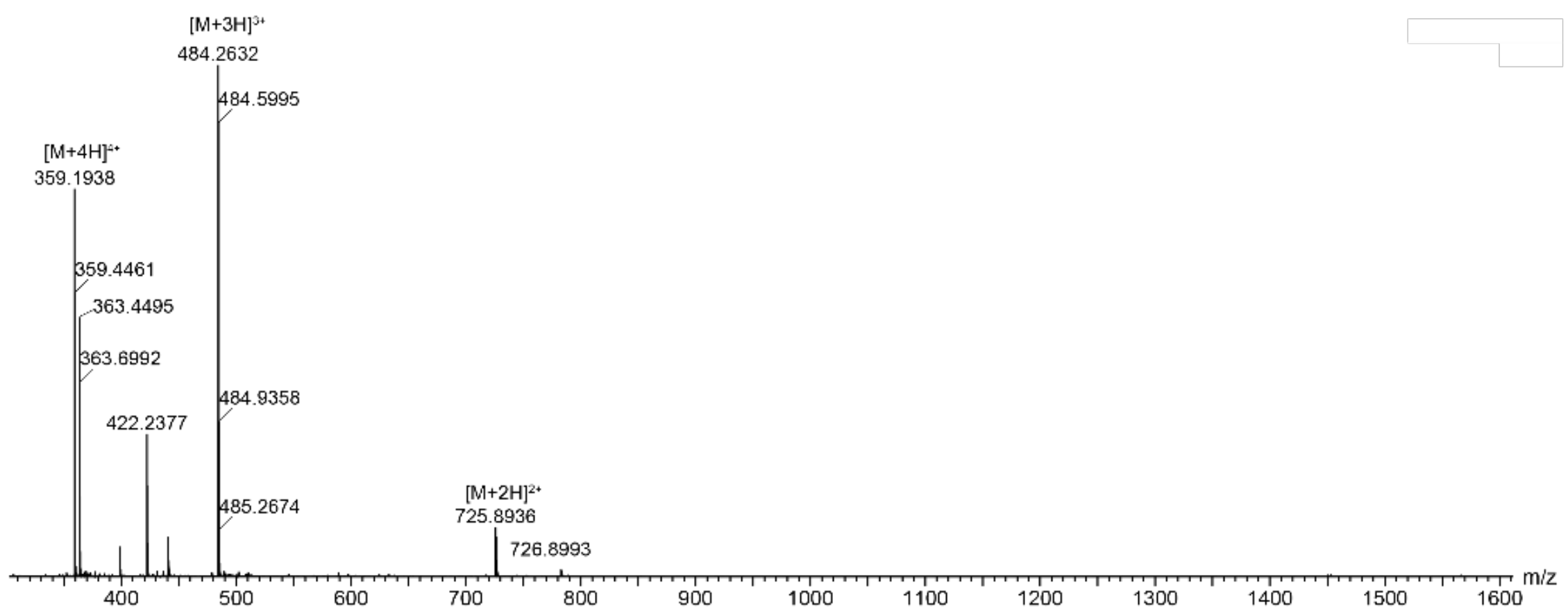

Figure S21: High resolution mass spectrum for peptide G2-3.

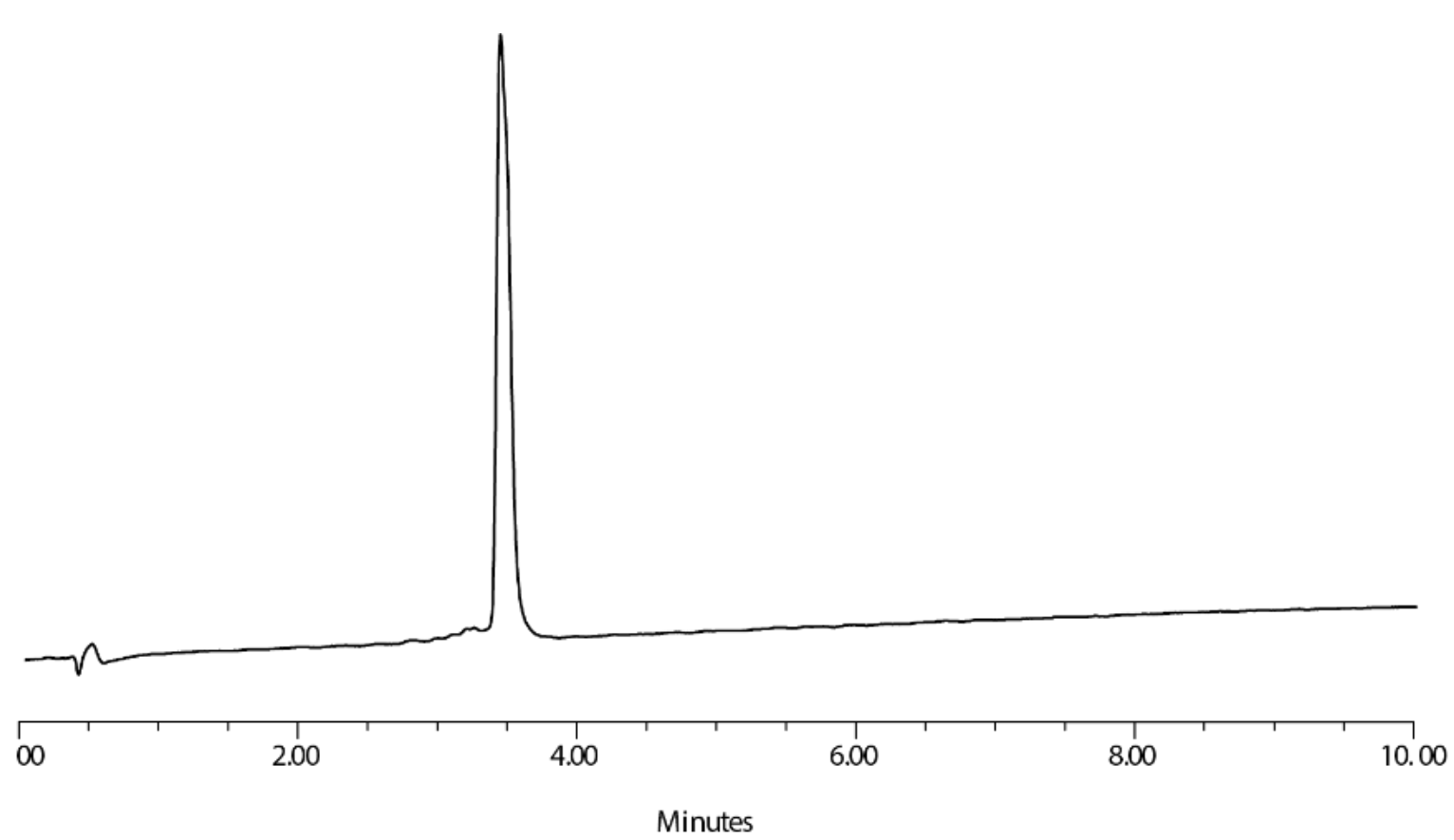

Figure S22: RP-HPLC chromatogram (gradient from 10 to 50% acetonitrile in water over 10 min at 2 ml/min, detection at 214 nm) for peptide G2-3.

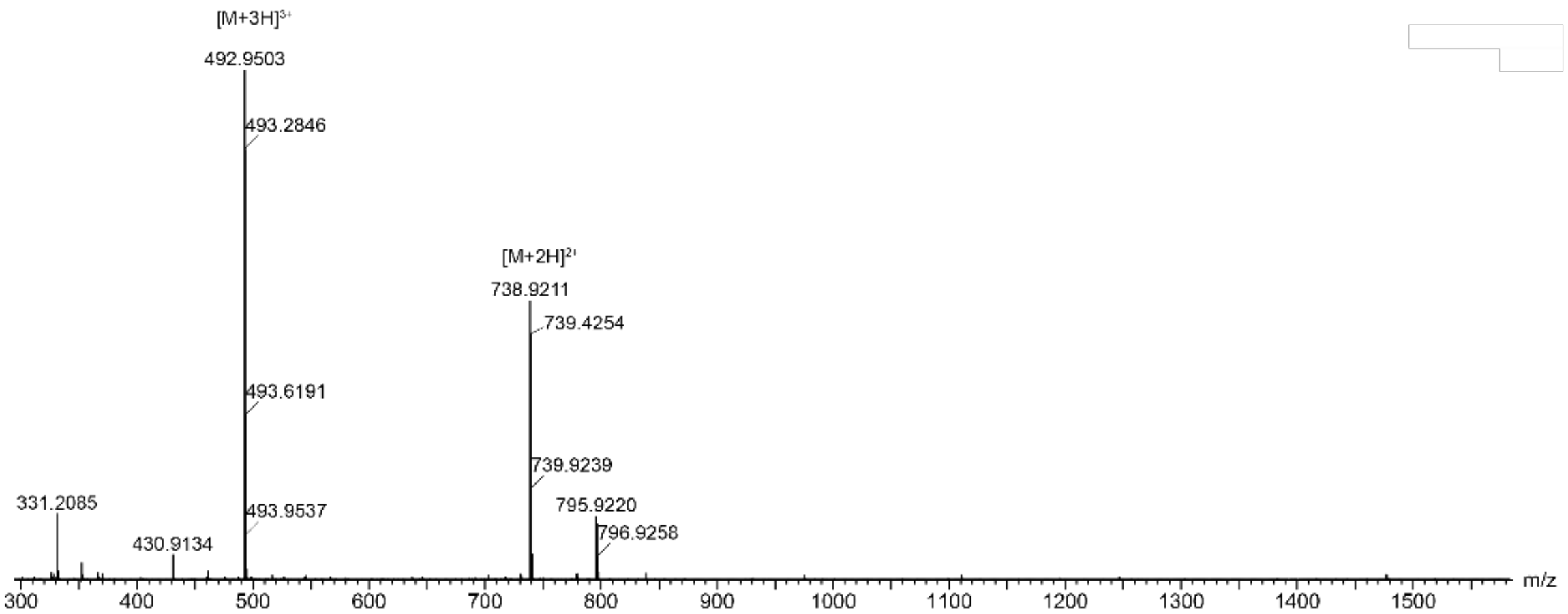

Figure S23: High resolution mass spectrum for peptide G2-4.

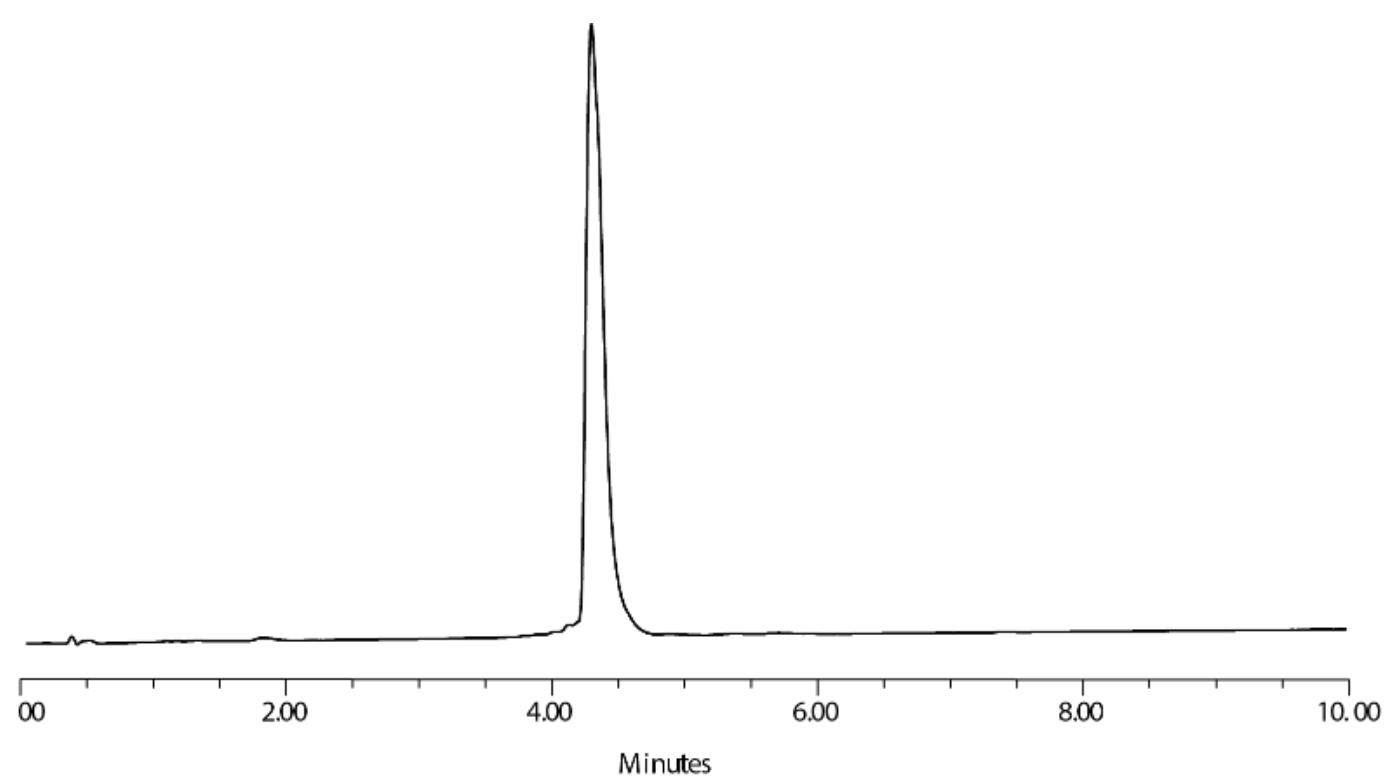

Figure S24: RP-HPLC chromatogram (gradient from 10 to 50% acetonitrile in water over 10 min at 2 ml/min, detection at 214 nm) for peptide G2-4.

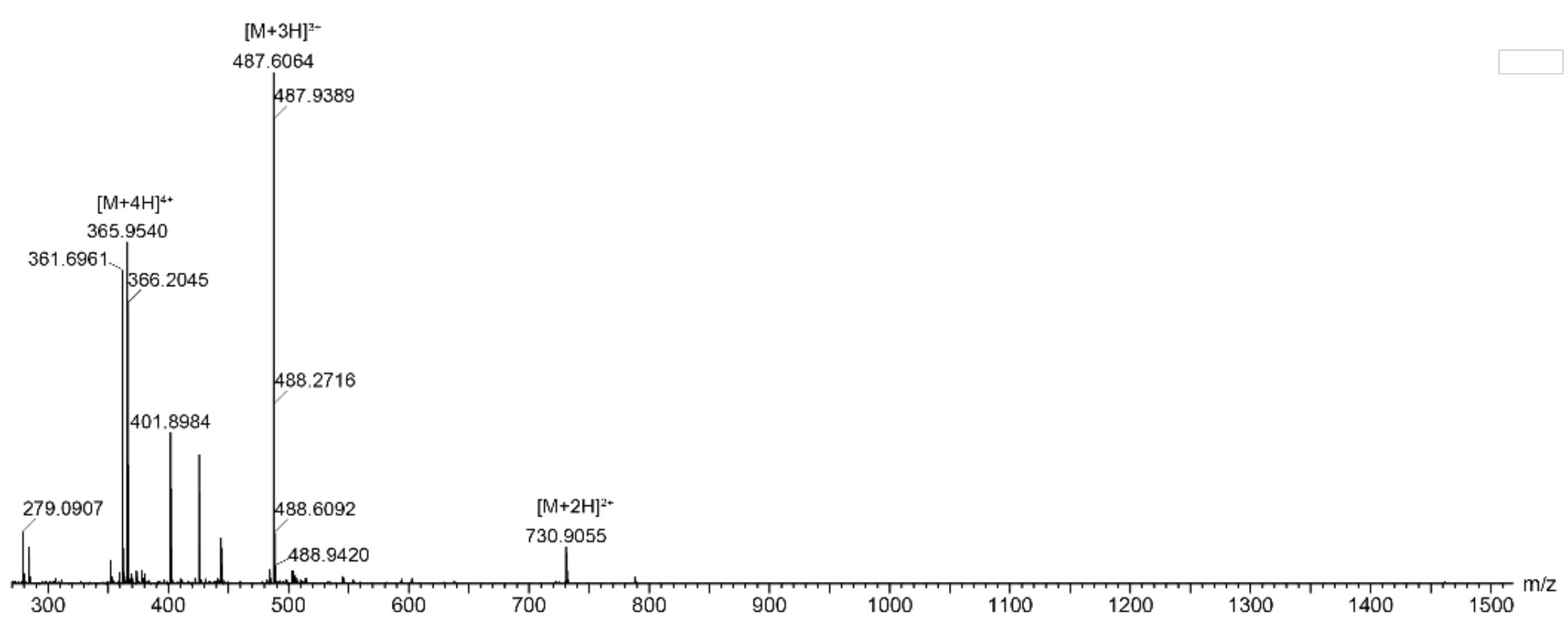

Figure S25: High resolution mass spectrum for peptide G2-5.

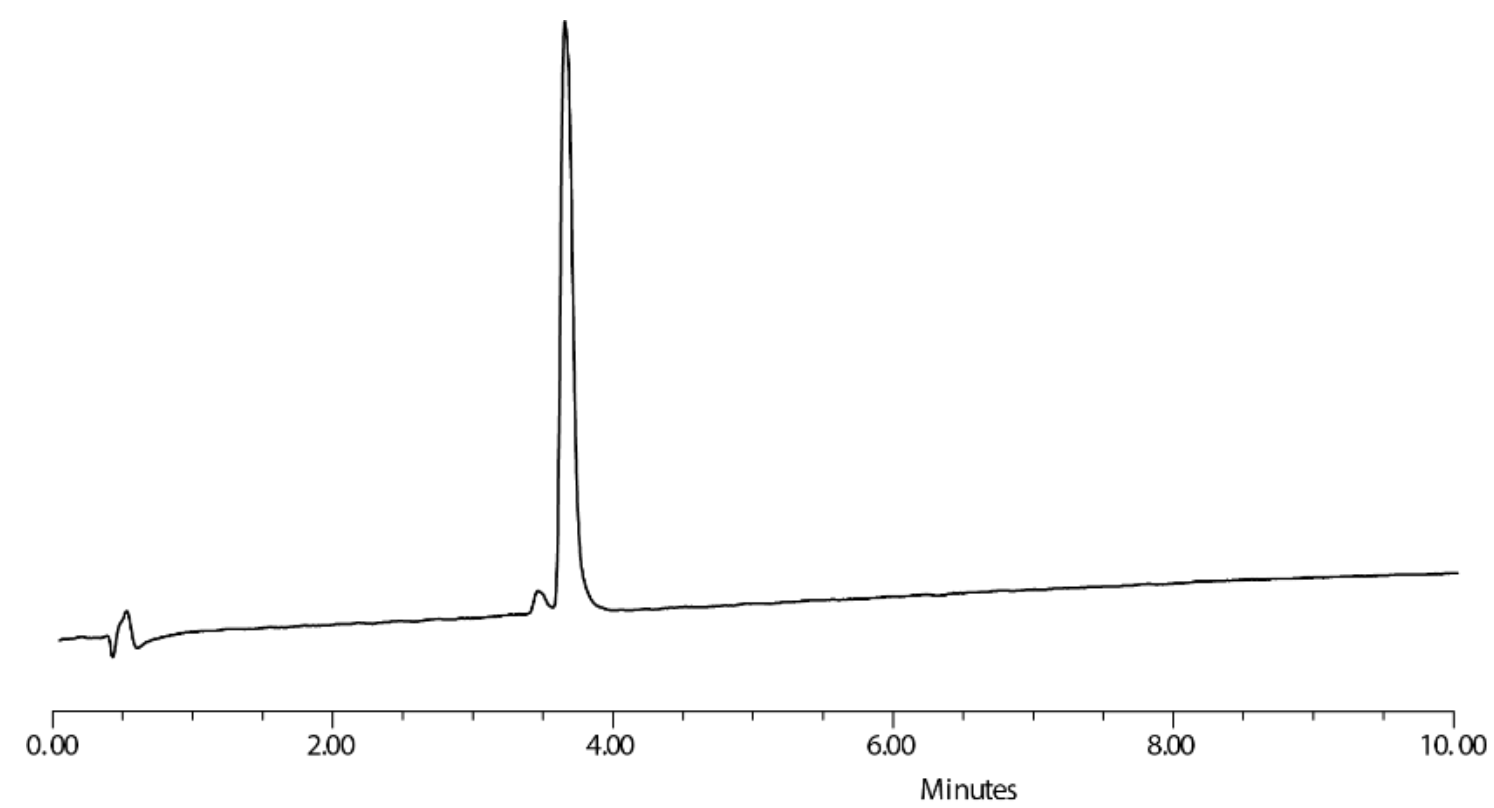

Figure S26: RP-HPLC chromatogram (gradient from 10 to 50% acetonitrile in water over 10 min at 2 ml/min, detection at 214 nm) for peptide G2-5.

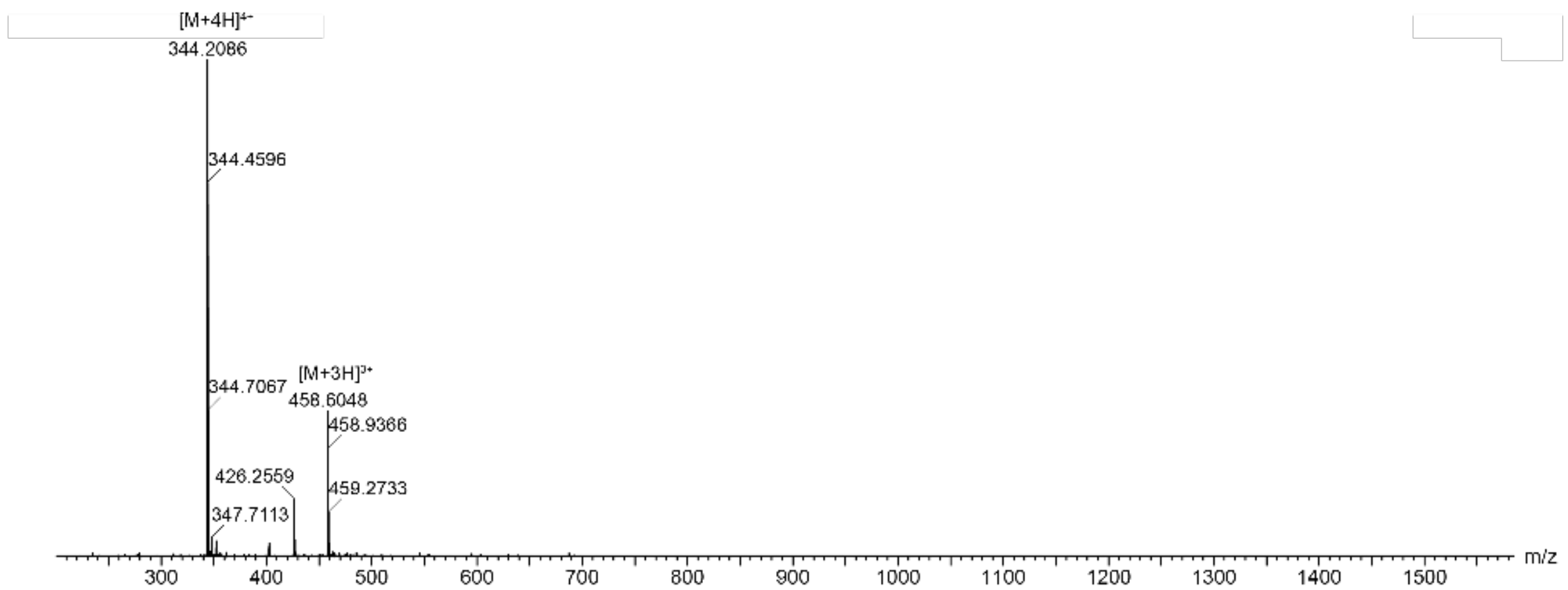

Figure S27: High resolution mass spectrum for peptide G2-6.

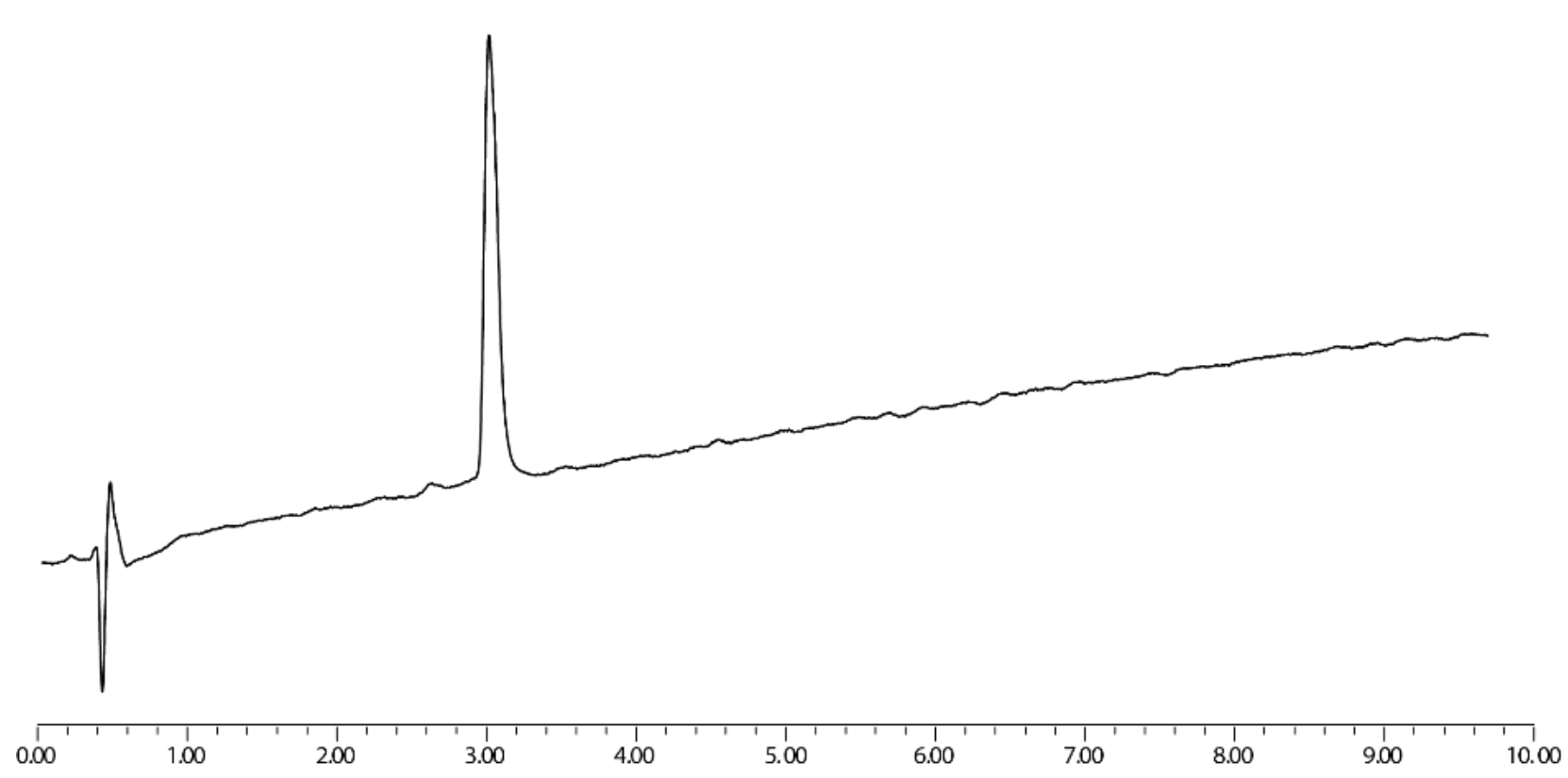

Figure S28: RP-HPLC chromatogram (gradient from 10 to 50% acetonitrile in water over 10 min at 2 ml/min, detection at 214 nm) for peptide G2-6.

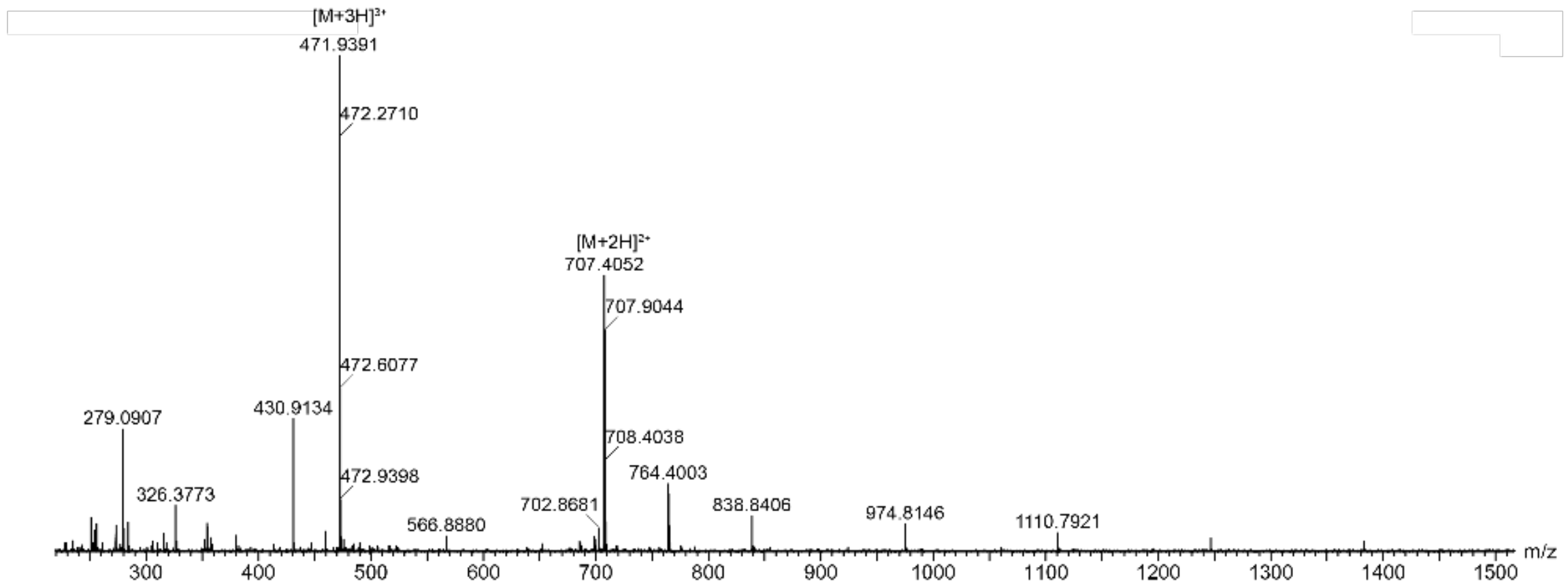

Figure S29: High resolution mass spectrum for peptide G2-7.

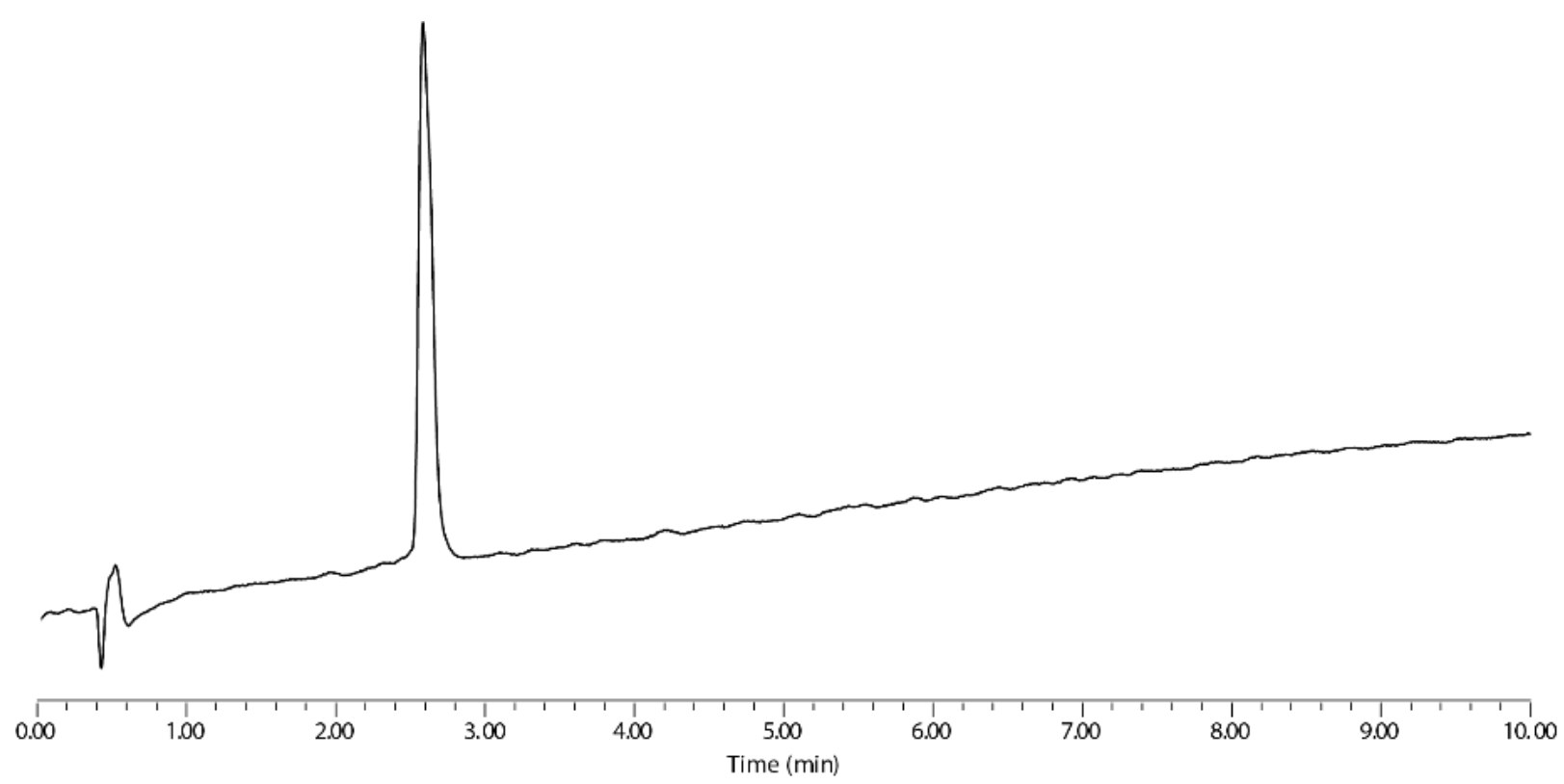

Figure S30: RP-HPLC chromatogram (gradient from 10 to 50% acetonitrile in water over 10 min at 2 ml/min, detection at 214 nm) for peptide G2-7.

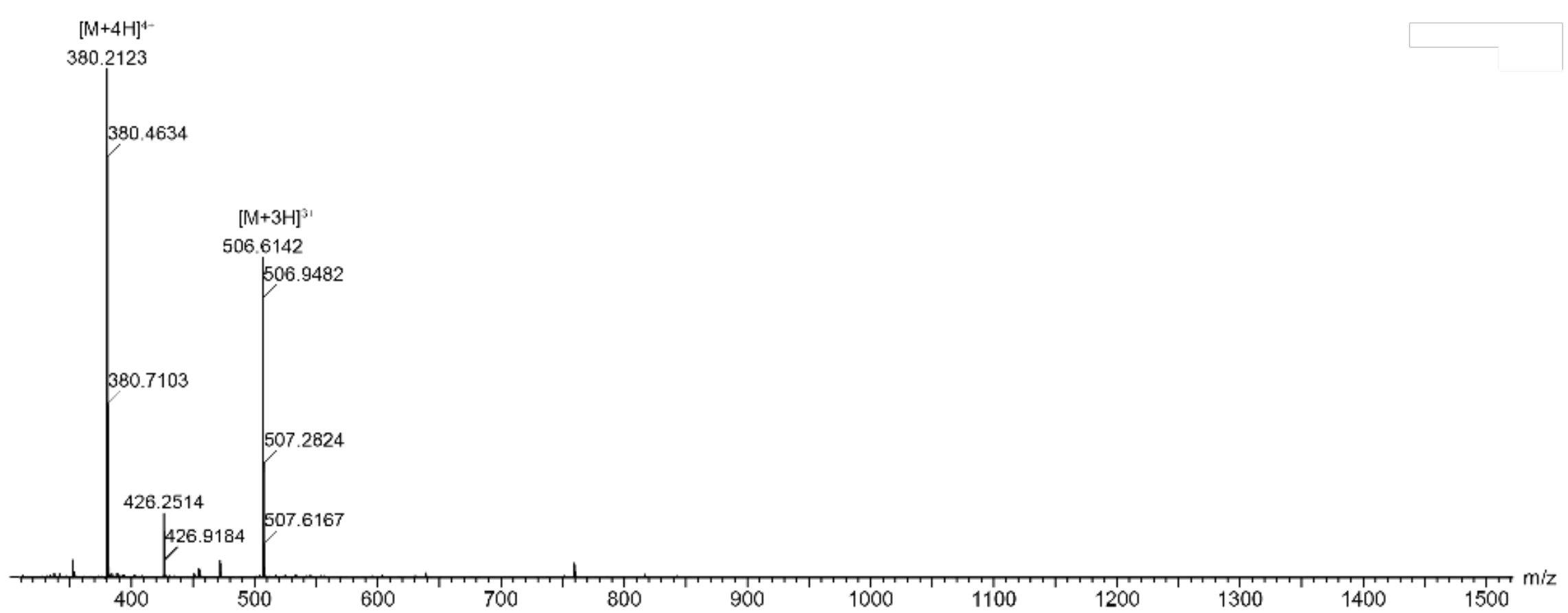

Figure S31*:* High resolution mass spectrum for peptide G2-8.

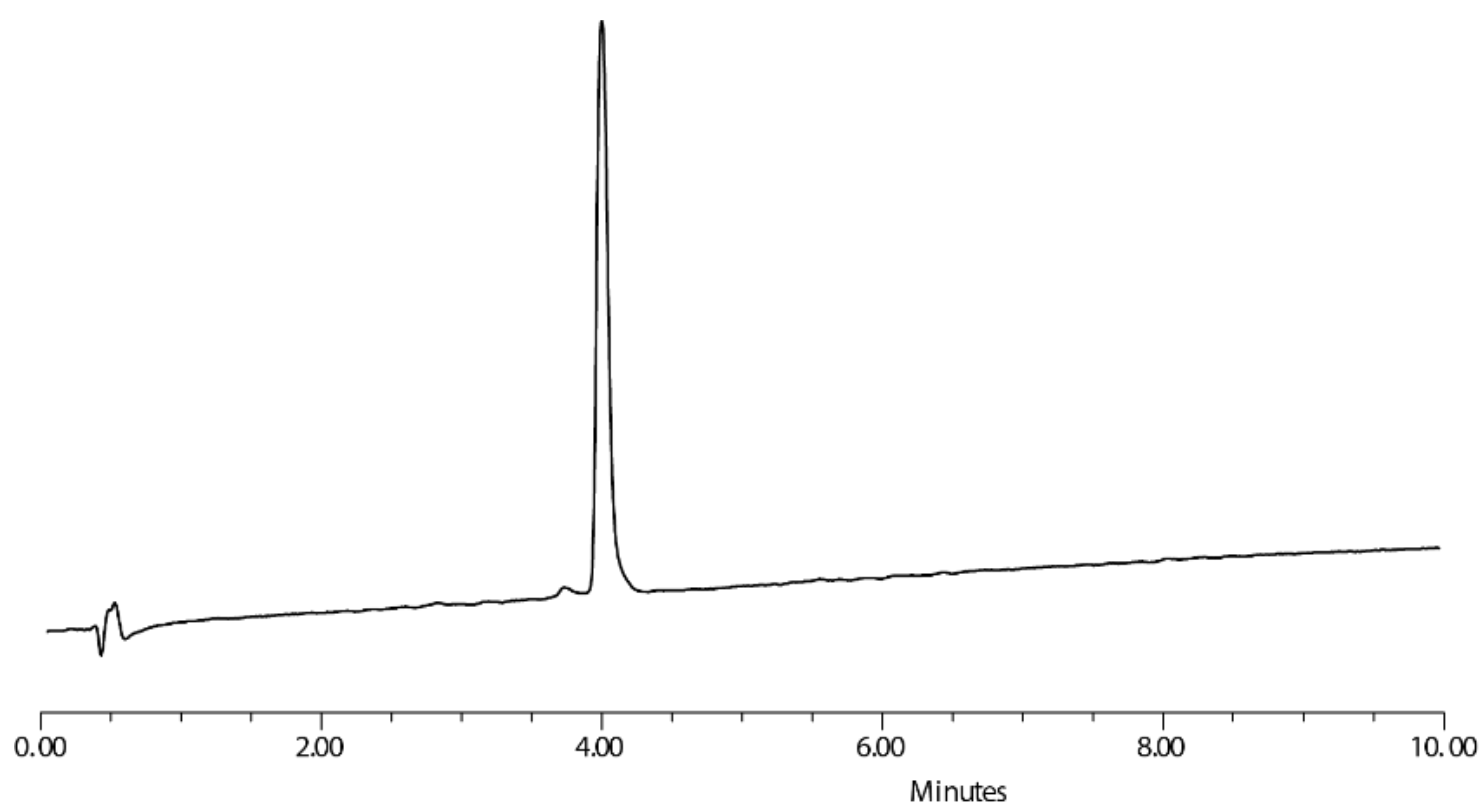

Figure S32: RP-HPLC chromatogram (gradient from 10 to 50% acetonitrile in water over 10 min at 2 ml/min, detection at 214 nm) for peptide G2-8.

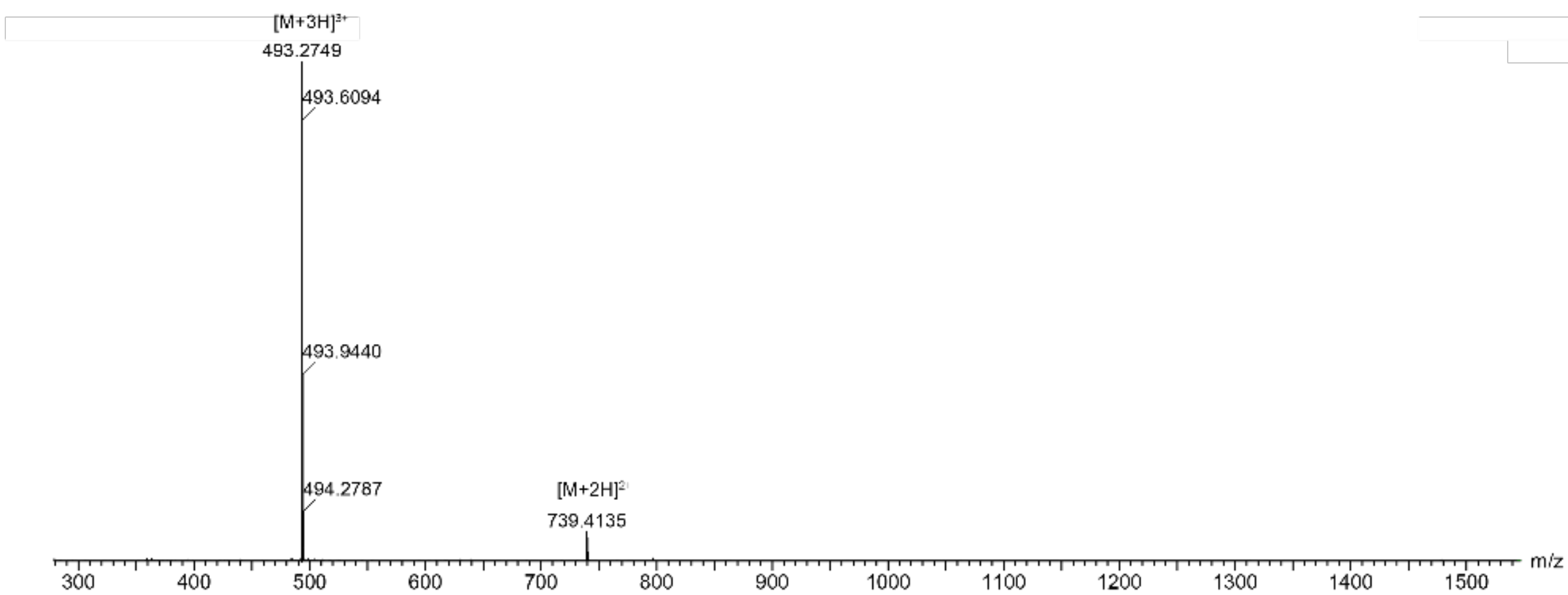

Figure S33: High resolution mass spectrum for peptide G2-9.

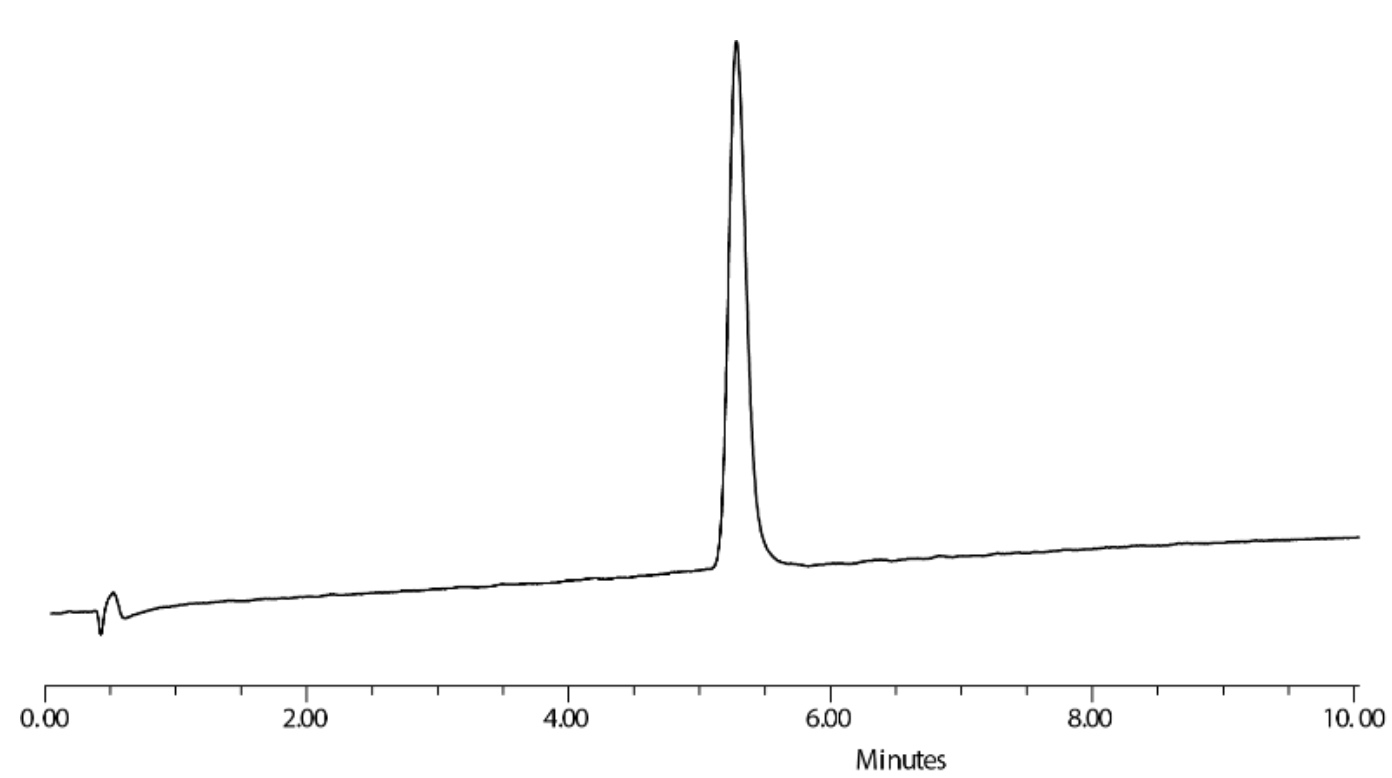

Figure S34: RP-HPLC chromatogram (gradient from 10 to 50% acetonitrile in water over 10 min at 2 ml/min, detection at 214 nm) for peptide G2-9.

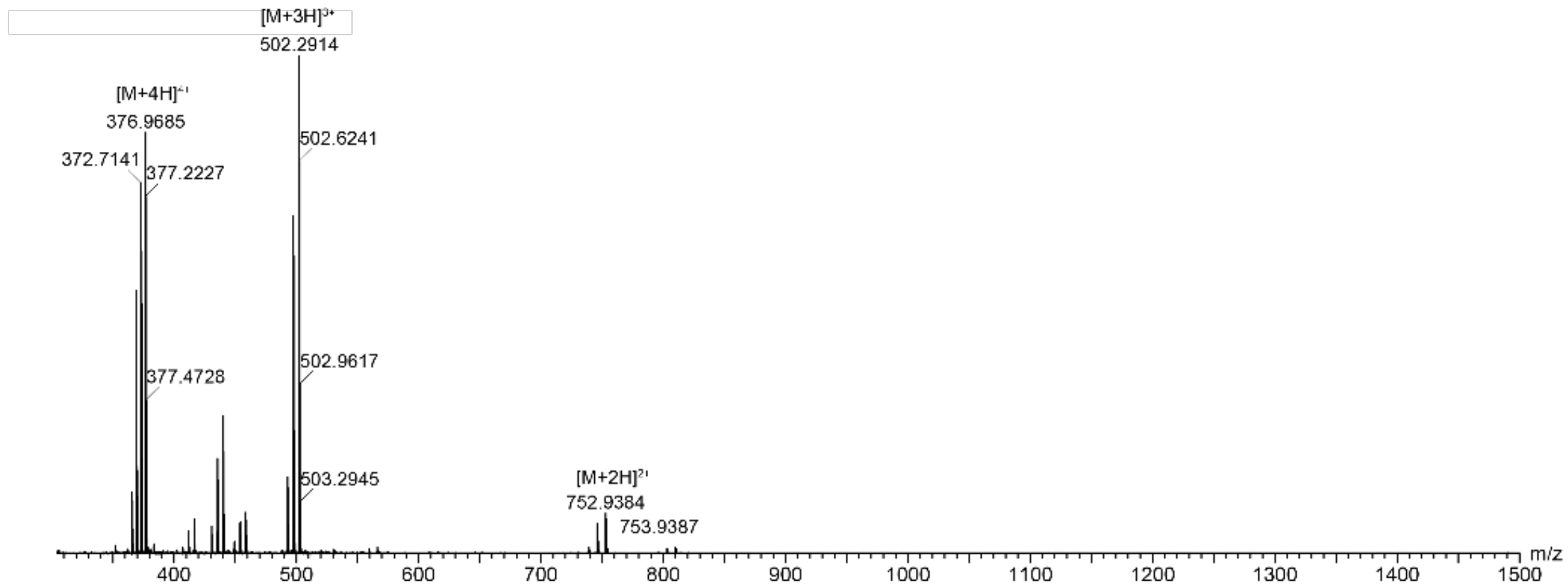

Figure S35: High resolution mass spectrum for peptide G2-10.

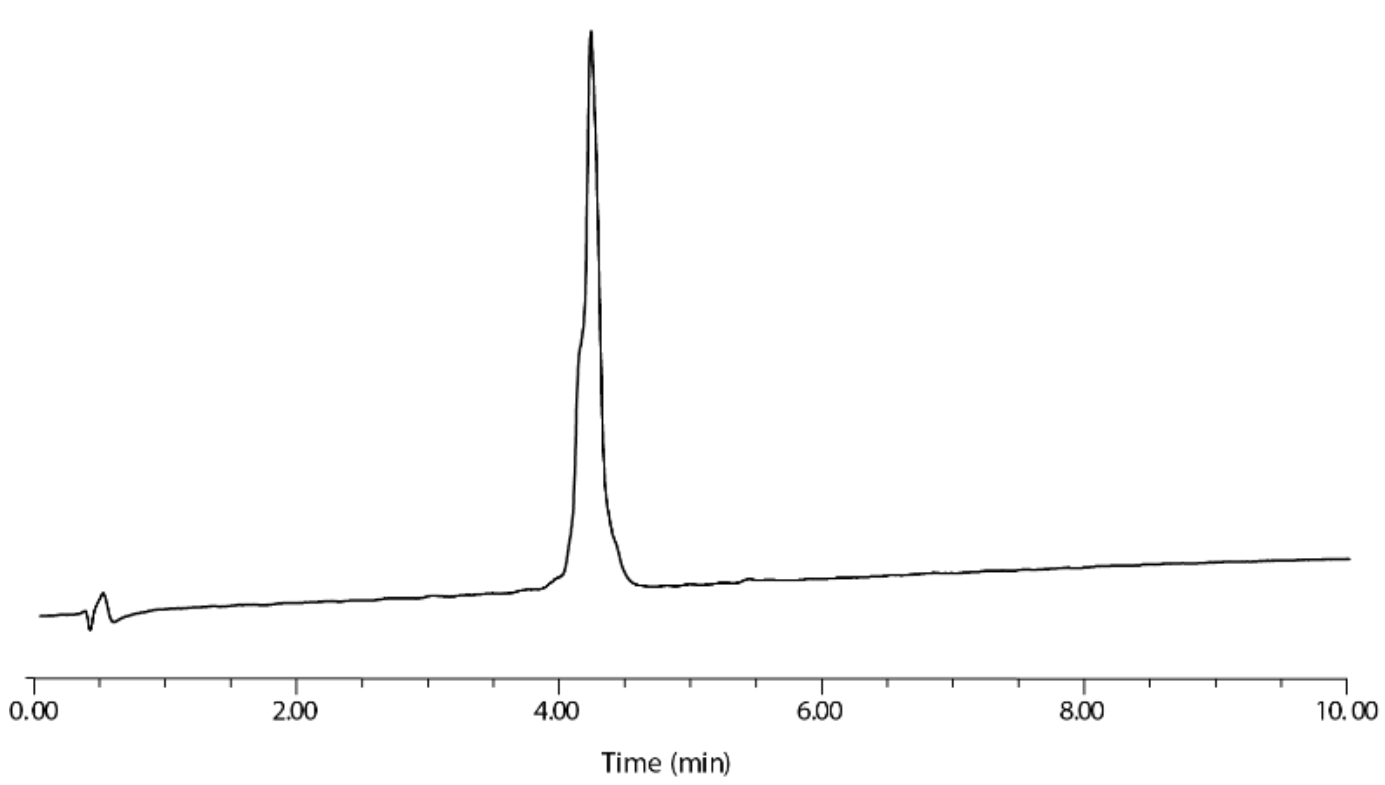

Figure S36: RP-HPLC chromatogram (gradient from 10 to 50% acetonitrile in water over 10 min at 2 ml/min, detection at 214 nm) for peptide G2-10.

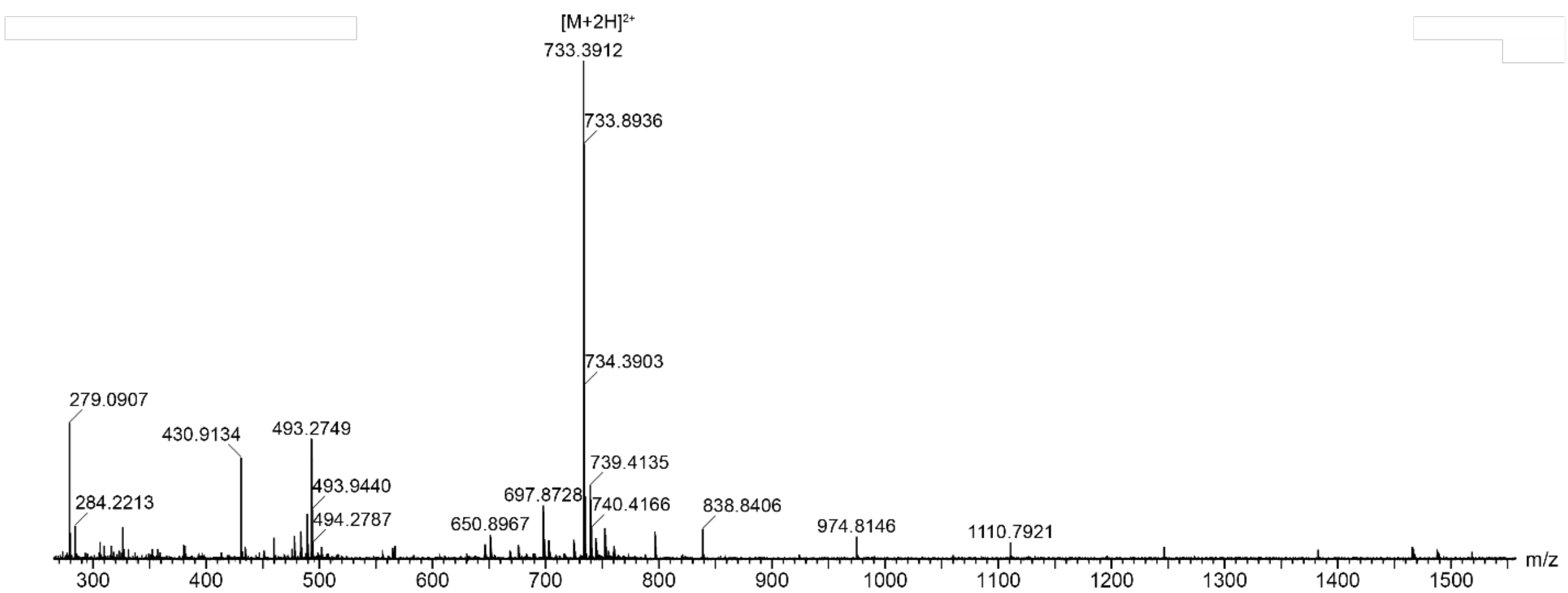

Figure S37: High resolution mass spectrum for peptide G2-11.

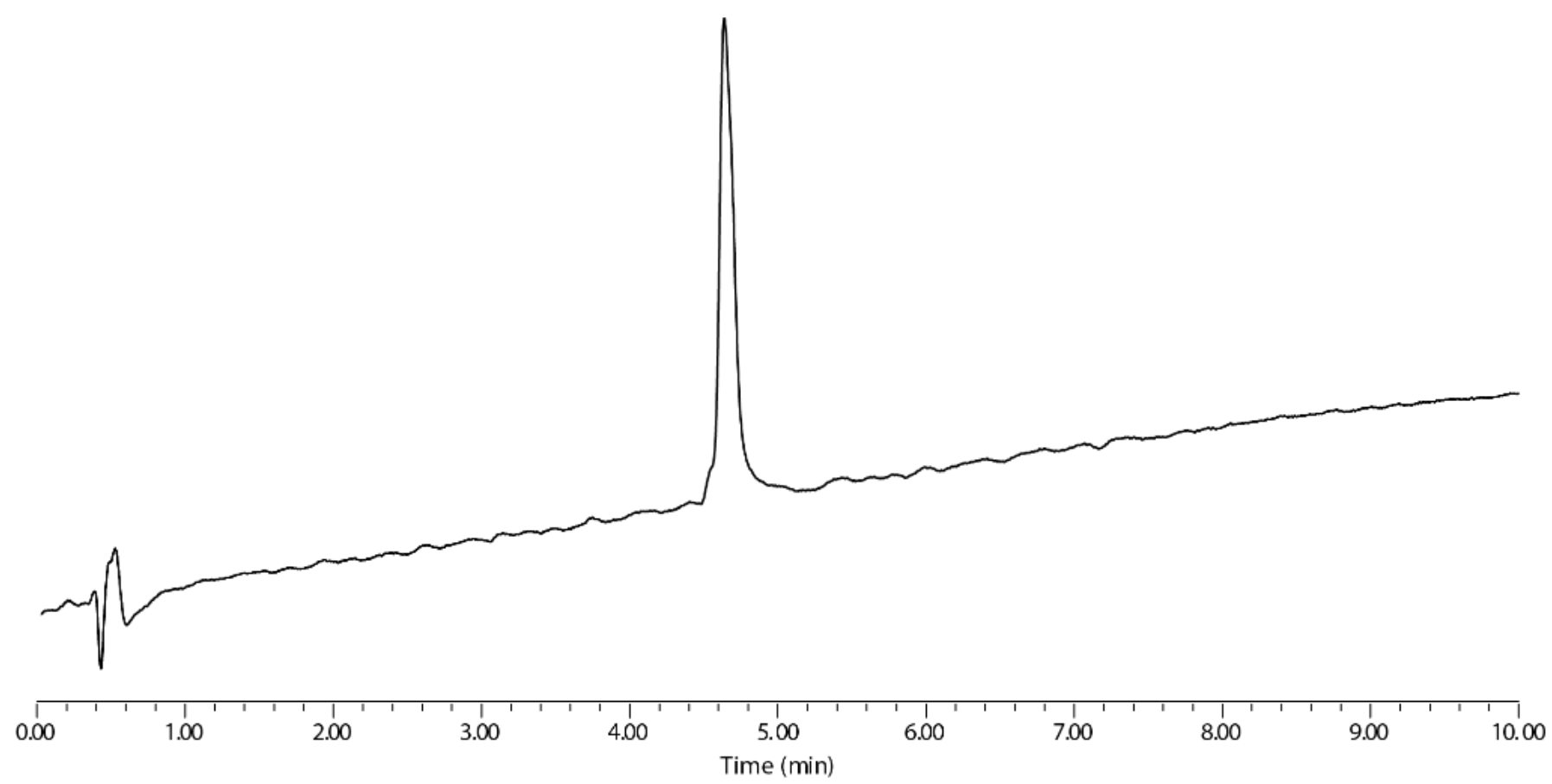

Figure S38: RP-HPLC chromatogram (gradient from 10 to 50% acetonitrile in water over 10 min at 2 ml/min, detection at 214 nm) for peptide G2-11.

CD deconvolution and spectra

Table S2: Estimated secondary structure content (%) using Beta Structure Selection (BeStSel) freely accessible webserver. (BeStSel - Protein Circular Dichroism Spectra Analysis)

	G1-8		G2-1		G2-2		G2-4		G2-10	
**Secondary Structure**	water	TFE*	water	TFE	water	TFE	water	TFE	water	TFE
**Helix**	0	75.3	0	66.2	10.1	93.5	0.6	80.1	10.4	100
**Antiparallel**	51.8	0	39.6	0	28.6	0	32.9	0.5	35.8	0
**Parallel**	0	24.7	0	32.9	0	0	0	0	0	0
**Turn**	0	0	0	0	0	0	0	0	0	0
**Others**	48.2	0	60.4	0.9	61.3	6.5	66.5	19.4	53.8	0

* 2,2,2-trifluoroethanol

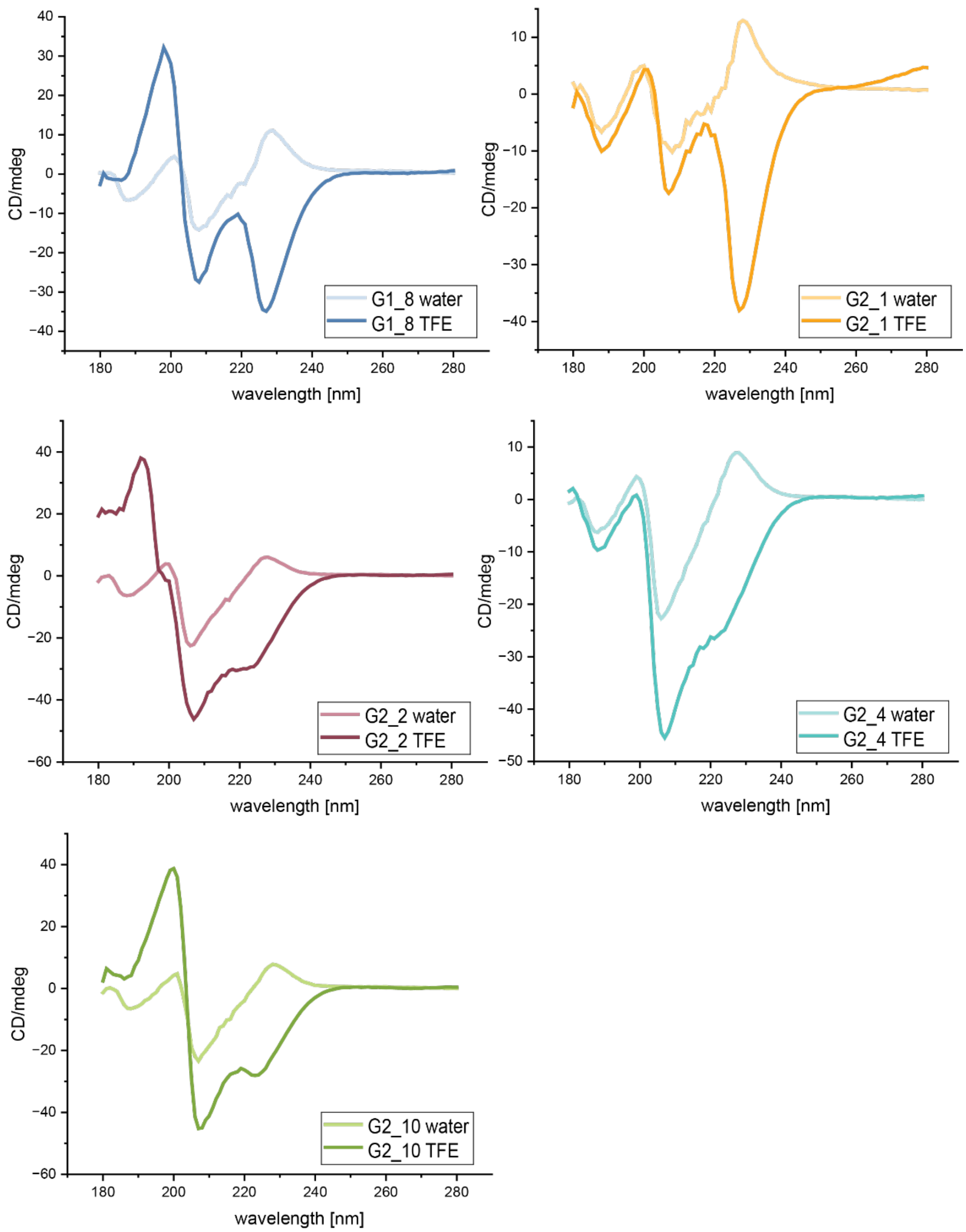

Figure S39: CD spectra measured at 50 μM peptide concentration.

Solution NMR studies

Table S3: Energy and structural ensembles of synthesized peptides.

**Variables**	**Peptide**				
	**G1-8**	**G2-1**	**G2-2**	**G2-4**	**G2-10**
Distance restrains, n#	8	2	4	7	4
Structure statistics, kj/mol					
Violations					
Total restrain violation energy	7.52	0.94	4.01	11.69	6.03
Distance restrains	7.52	0.94	4.01	11.69	6.03
Total Force field energy (YASARA)	-6869.18	-7113.77	-6948.50	-6708.85	-8766.51
Internal solute energy	-2120.16	-2251.678	-1952.98	-1962.11	-613.26
Electrostatic solv. Energy	-4833.14	-4941.029	-5078.63	-4860.93	-8648.29
Van der Waals solv. Energy	84.12	78.937	83.11	114.185	495.04
Mean RMSD* (Å), mean±SD					
Backbone global	1.64 ± 0.62	2.23 ± 0.66	2.12 ± 0.57	0.94 ± 0.18	1.26 ± 0.52
Heavy atoms	2.43 ± 0.61	3.32± 0.82	3.38 ± 0.80	1.87 ± 0.35	2.73 ± 0.65

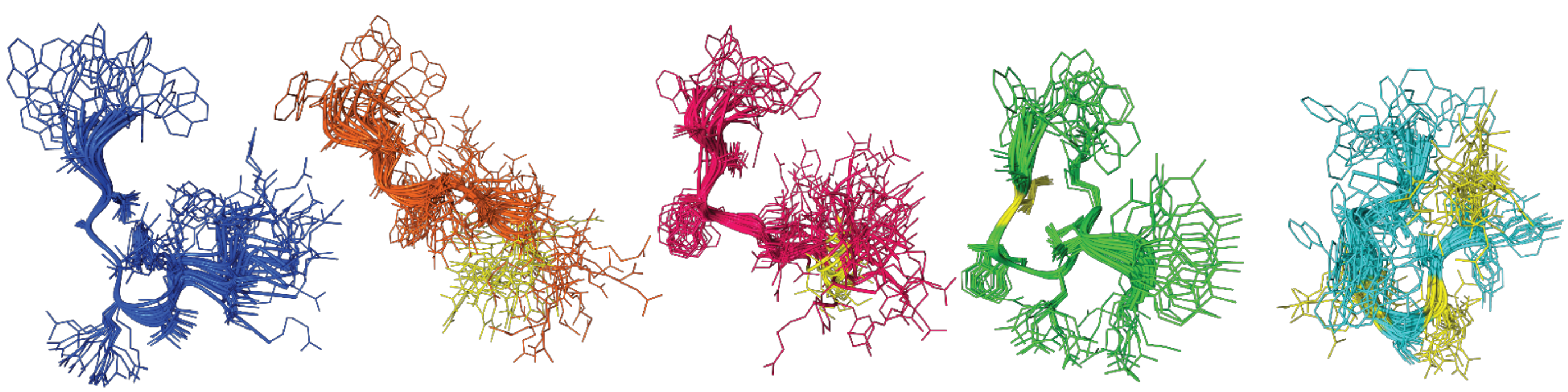

G1-8 G2-1 G2-2 G2-4 G2-10

Figure S40: Solution NMR structural ensembles of the selected peptide analogues G1-8, G2-1, G2-2, G2-4 and G2-10 (from left to right). The 20 lowest energy structures obtained from NOE-restrained structure calculations are superimposed and displayed as backbone traces. Ensembles are aligned over the ordered peptide region to visualize the conformational distribution and local structural flexibility. Residues deviating from G1-8 are colored in yellow.

Fluorescence Displacement Assay DAPI

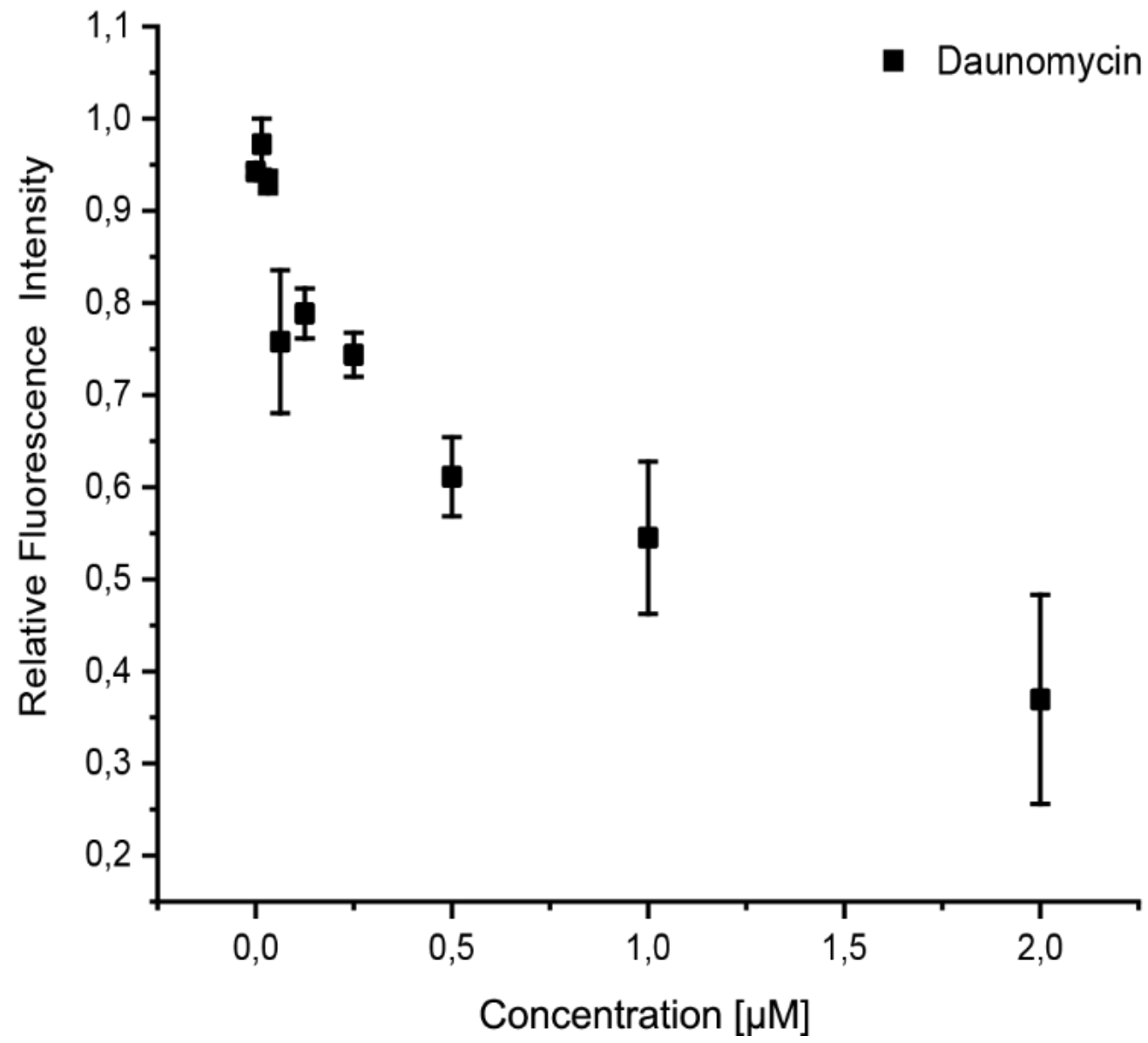

Figure S41: Displacement of DAPI from plasmid DNA. Peak fluorescence (450 nm for DAPI) is normalized to the highest value of fluorescence intensity. Lower fluorescence indicates dye displacement. DAPI to DNA phosphate ratio is 0.025. Daunomycin measured from 2 µM in a 2-fold dilution series. Data points represent average values from two measurements, with standard deviation indicated by the error bar.
